# Proteogenomic discovery of *RB1*-defective phenocopy in cancer predicts disease outcome, response to treatment, and therapeutic targets

**DOI:** 10.1126/sciadv.adq9495

**Published:** 2025-03-26

**Authors:** Jacopo Iacovacci, Rachel Brough, Fatemeh Ahmadi Moughari, John Alexander, Harriet Kemp, Andrew N. J. Tutt, Rachael Natrajan, Christopher J. Lord, Syed Haider

**Affiliations:** ^1^ The Breast Cancer Now Toby Robins Research Centre, The Institute of Cancer Research, London SW3 6JB, UK.; ^2^ Data Science Unit, Fondazione IRCCS Istituto Nazionale dei Tumori di Milano, Milano 20133, Italy.; ^3^ CRUK Gene Function Laboratory, The Institute of Cancer Research, London SW3 6JB, UK.

## Abstract

Genomic defects caused by truncating mutations or deletions in the Retinoblastoma tumor suppressor gene (*RB1*) are frequently observed in many cancer types leading to dysregulation of the RB pathway. Here, we propose an integrative proteogenomic approach that predicts cancers with dysregulation in the RB pathway. A subset of these cancers, which we term as “RBness,” lack *RB1* genomic defects and yet phenocopy the transcriptional profile of *RB1*-defective cancers. We report RBness as a pan-cancer phenomenon, associated with patient outcome and chemotherapy response in multiple cancer types, and predictive of CDK4/6 inhibitor response in estrogen-positive breast cancer. Using RNA interference and a CRISPR-Cas9 screen in isogenic models, we find that RBness cancers also phenocopy synthetic lethal vulnerabilities of cells with *RB1* genomic defects. In summary, our findings suggest that dysregulation of the RB pathway in cancers lacking *RB1* genomic defects provides a molecular rationale for how these cancers could be treated.

## Teaser

A proteogenomic approach identifies a class of cancers (*RBness*) that phenocopies *RB1-*defective tumors.

## INTRODUCTION

*RB1* is a prototypic tumor suppressor gene that is frequently mutated and/or deleted in a variety of human cancers (*1*). *RB1*’s canonical role is in the regulation of the cell cycle, a function that is primarily mediated by its control over the E2F family of transcription factors (*2*). The Rb protein recruits chromatin regulators via the LxCxE-binding cleft in its pocket domain, which in turn controls the expression of E2F target genes (*3*). The LxCxE motif that enables interactions with histone deacetylases (HDACs) and chromatin modelers is independent of Rb’s E2F binding domain and therefore adds further complexity to E2F-mediated functionality of Rb (*4*). For instance, deacetylation of histones by HDACs is necessary for the Rb-mediated repression of genes activated in G_1_ cell cycle. Furthermore, Rb also binds to histone methyltransferases and histone demethylases, suggesting an epigenetic role in the maintenance of heterochromatin organization and stability (*4*). In summary, the Rb protein complex is multifunctional (*2*) and regulates a plethora of biological processes from chromatin regulation and stability, senescence, apoptosis, and mitochondrial function (*1*, *5*, *6*). In part, at least, the loss of Rb function in tumor cells results in augmented cell proliferation due to dysregulation of the G_1_-S cell cycle checkpoint (*1*, *7*).

From a clinical perspective, *RB1* deleterious mutations and deletions are associated with poor overall survival (OS) and response to chemotherapy in cancers such as those of the breast, lung, and prostate (*8*). For example, in triple-negative breast cancer (TNBC), a highly aggressive subtype with poor survival properties (5-year OS rate of 77%), approximately 13% of tumors harbor mutations or deletions of the *RB1* gene. Similarly, mutational and structural defects in *RB1* are frequently observed in many cancers accounting for up to 25% of bladder cancer (BLCA) and sarcomas (*9*). Recent studies have also demonstrated that *RB1* mutation causes clinical resistance to inhibition of cyclin-dependent kinases 4 and 6 (CDK4/6) (*10*–*12*), and in preclinical studies, *RB1* mutation, deletion, or loss of protein expression are associated with sensitivity to inhibition of either PLK1, CHK1, SKP2, AURKA, or AURKB (*13*–*15*). Given the clinical implications of *RB1* status in cancer (*8*, *16*), a number of molecular signatures that define the functional state of *RB1* have been proposed (*17*–*20*). Chen *et al.* (*17*) identified a transcriptomic RB loss signature (RBS) associated with *RB1* genomic alterations in pan-cancer cell lines. Ertel *et al.* (*18*) also identified a transcriptomic RB loss signature by aggregating genes up-regulated by *RB1* deletion in fibroblastic models or murine liver and genes repressed by the activation of *RB1* in cell lines. Malorni *et al.* (*19*) proposed a transcriptomic *RB1* loss-of-function signature (RBsig) capturing genes correlated with *E2F1* and *E2F2* gene expression in breast cancer cell lines. Knudsen *et al.* (*20*) derived a gene expression–based RB-pathway activity signature in *RB1*-deleted isogenic breast cancer cell lines exposed to CDK4/6 inhibitors, thereby modulating RB phosphorylation and resulting in inactivation of *RB1*. While these signatures have been tested in clinical samples, there are three key limitations of these signatures. First, none of these signatures take into account instances where *RB1* protein is reduced or hyperphosphorylated in the absence of *RB1* mutation or *RB1* deletion. Second, the discovery of these signatures is primarily influenced by cell line models, and it remains to be elucidated whether the signatures identified in patient samples would capture additional sources of clinical heterogeneity. Third, it remains unclear whether existing molecular signatures of *RB1* status are associated with response to chemotherapy in adequately powered clinical studies. To address these limitations, we describe here a proteogenomic approach that allows the identification of *RB1*-defective cancers as well as cancers that phenocopy *RB1*-defective cancers, a class of cancers we term as “RBness.” Part of our reason for defining RBness cancers is that these are likely to behave in much the same way as *RB1* mutant cancers and could be treated in a similar way. By analogy, BRCAness cancers, i.e., those that share molecular features of germline *BRCA1* or *BRCA2* mutated cancers (gBRCAm), also phenocopy the platinum-salt or poly(adenosine diphosphate–ribose) polymerase (PARP) inhibitor sensitivity seen in gBRCAm cancers (*21*–*23*). In defining RBness, we find that it is common in cancers such as of the breast and ovary, that it is associated with aggressive forms of the disease, and that it is associated with outcome in chemotherapy-treated patients. We also show that RBness cancers have a transcriptional program that is correlated with genes that are known to be synthetic lethal with *RB1* mutation, suggesting new ways these cancers that phenocopy *RB1* defects could be treated.

## RESULTS

### Integration of tumor proteogenomic data identifies an RBness subtype of breast cancer that exists in the absence of *RB1* genomic alterations

**Fig. 1.**
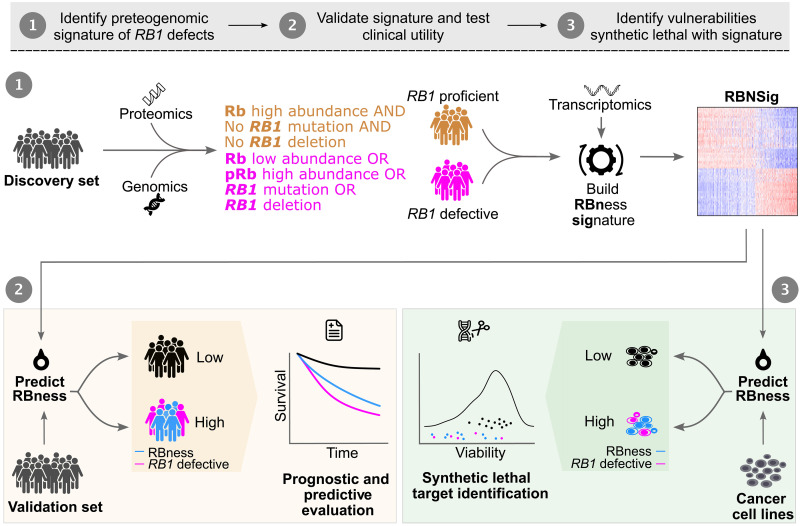
Study overview. Schematic depicting a proteogenomic computational approach for the discovery and validation of mRNA phenocopy of *RB1*-defective cancers using multiomic molecular profiles. Constituent genes of RBNSig were interrogated for association with the clinical outcome and therapeutic response and for the identification of candidate therapeutic vulnerabilities. For details, see Materials and Methods: Discovery of RBNSigs.

**Fig. 2.**
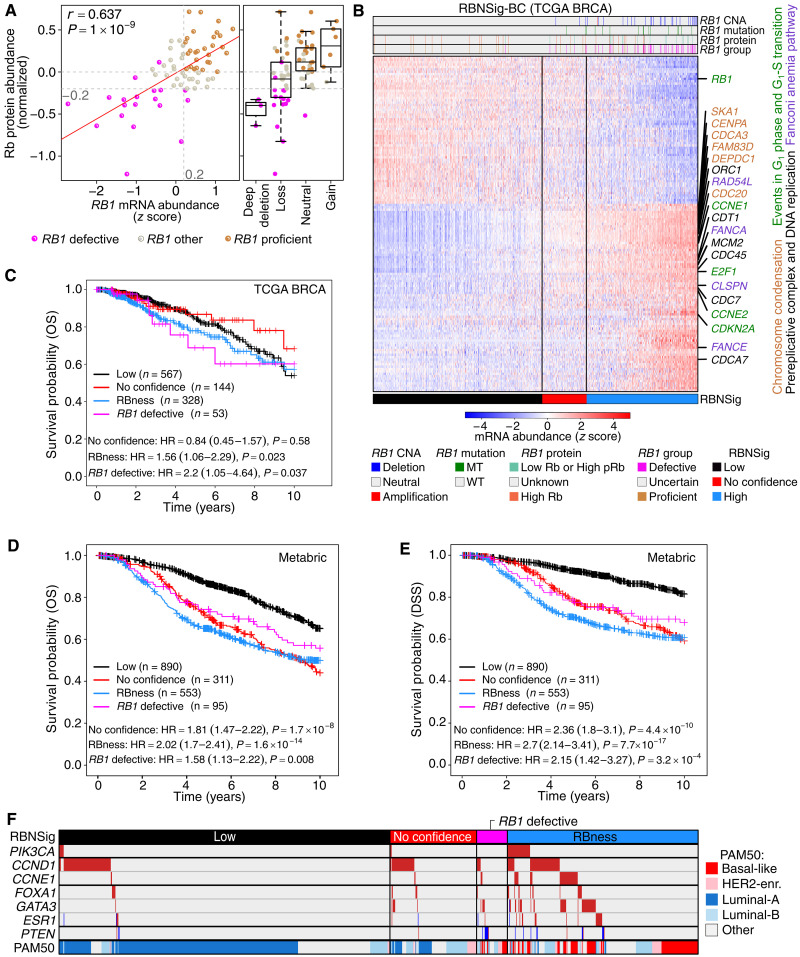
RBNSig of breast cancer. (**A**) Rb protein abundance as a function of *RB1* mRNA abundance *z* score (scatter plot) and *RB1* copy number alteration (CNA) across the 74 TCGA BRCA samples (discovery set) for which CPTAC proteomics data were available. (**B**) The RBNSig of breast cancer (BC) along with predicted RBNSig-BC groups (low, no confidence, and high) is shown for all samples of TCGA’s breast cancer cohort (1093 patients). Vertical black lines in the heatmap show patients’ stratification into three RBNSig-BC groups: high (>55% signature alignment; RBNSig, blue), low (<45% signature alignment; RBNSig, black), no confidence (45 to 55% alignment; RBNSig, red). “*RB1* CNA” indicates copy number states: amplification, neutral, and deep deletion. “*RB1* mutation” indicates mutation status: MT, truncating mutation; WT, nontruncating mutation; or wild type. “*RB1* protein” indicates low Rb abundance or high phosphorylated Rb (pRb) relative to total Rb. “*RB1* status” indicates *RB1*-defective and *RB1*-proficient status inferred from CNA, mutation, or proteomic profiles. (**C**) Prognostic assessment (OS) of RBNSig-BC groups. The RBNSig-BC–low group was treated as the reference group in a multivariable Cox proportional hazards model adjusted for age, tumor size (T-stage), and lymph node status. The *RB1*-defective group (*n* = 53) represents samples with known *RB1* defects from both the RBNSig-BC–low and RBNSig-BC–high groups. (**D** and **E**) Prognostic assessment of RBNSig-BC in Metabric cohort using OS (D) and DSS (E). Multivariable Cox proportional hazards model was adjusted for age, tumor size (T-stage), and lymph node status. (**F**) Breast cancer driver gene enrichment in RBNSig-BC groups. Columns represent patients, and rows indicate CNA status: amplifications (red) and deletions (blue). PAM50 classification “Other” (gray) include both normal-like and unclassified samples.

To identify cancers that phenocopy *RB1* genomic defects (deleterious mutations or deletions), we integrated proteomic, transcriptomic, and genomic data from 1093 breast tumors described in The Cancer Genome Atlas’ (TCGA) breast cancer (BRCA) dataset (Fig. 1) (*24*). First, we defined high-confidence *RB1*-defective (*n* = 19) and -proficient (*n* = 28) samples using both the protein abundance and phosphorylation status of Rb (pRb) in TCGA’s Clinical Proteomic Tumor Analysis Consortium (CPTAC) mass spectrometry proteomic dataset (Materials and Methods: Discovery of RBNSigs). TCGA mass spectrometry protein abundance data were preferred since these data demonstrated higher correlation with mRNA abundance (Pearson’s *r* = 0.637, *P* = 1 × 10^−9^; Fig. 2A) when compared to the reverse-phase protein microarray (RPPA) platform (Pearson’s *r* = 0.24, *P* = 6.9 × 10^−13^; fig. S1). Second, we extended the *RB1*-defective group by including additional 34 tumor samples harboring either an *RB1* deep deletion or *RB1* truncating mutation (MT). Samples with a deep deletion exhibited the lowest median Rb protein abundance, and, as expected, the Rb abundance was proportional on average to the number of *RB1* copies present in the tumor sample (Fig. 2A). Using this discovery set of high-confidence *RB1*-proficient and *RB1*-defective samples, a subsampling-driven robust differential mRNA abundance analysis was performed between these two groups resulting in an RBness signature (RBNSig) of breast cancer (BC) (fig. S2 and table S1; Materials and Methods: Discovery of RBNSigs). The RBNSig-BC was tested in the entire TCGA BRCA dataset (*n* = 1093; Fig. 2B and table S2) by classifying samples into RBNSig-high (signature alignment >55%, *n* = 375), RBNSig-low (signature alignment <45%, *n* = 569), and no confidence groups (signature alignment 45 to 55%, *n* = 149). As expected, the RBNSig-high group was enriched with samples displaying either an *RB1* deep deletion, an *RB1* MT, low Rb abundance, or high pRb [*n* = 47, odds ratio (OR) = 81.06, *P* < 2.2 × 10^−16^, Fisher’s exact test]. However, the RBNSig-high group also identified additional 328 patients lacking *RB1* genomic defects, hereafter termed an RBness group that represents a transcriptional phenocopy of genomically defective *RB1* cancers (Fig. 2B). We acknowledge that, if matched proteomic profiles were available, many of these patients with RBness in the TCGA breast cancer cohort could be explained by low Rb protein or hyperphosphorylated Rb. This observed fraction of RBness breast cancers raises an interesting question as to what fraction of the RBness population could be explained by obvious genomic or proteomic alterations in the *RB1* gene/protein and what fraction might have a different cause of RBness. To estimate this, we applied RBNSig-BC to TCGA breast cancers where matched DNA and protein profiling data were available (*n* = 74). RBNSig-BC called 33 samples as signature high. Of these, the majority (*n* = 23, 70%) had *RB1* disruption (either *RB1* mutation or copy number alteration, low Rb protein, or hyperphosphorylated Rb), with only 30% harboring RBness in the absence of a genomic or proteomic alteration in *RB1* itself. We further tested the reproducibility of RBNSig-BC–defined subtypes in an independent proteogenomic cohort of breast cancer (CPTAC 2/3, *n* = 122) (*25*). RBNSig-BC–predicted groups (low, no confidence, and high) showed a gene expression profile similar to that observed in the TCGA discovery data, with enrichment of *RB1*-defective cancers in RBNSig-BC–high group (OR = 4, *P* = 0.0016, Fisher’s exact test; fig. S3A); however, 16 low Rb patients were misclassified as RBNSig-BC–low (i.e., *RB1* proficient). The presence of these false negatives is likely due to a combination of factors including the predictive limitations of RBNSig-BC and the absence of thresholds for defining the functional state of Rb, which could affect the resulting ground truth.

To characterize RBNSig-BC, we evaluated the functional properties of the signature genes using the Local STRING network clusters (*26*). The STRING protein clusters revealed an enrichment for key hallmarks of Rb-associated dysregulation including (i) events in G_1_ phase and G_1_-S transition associated with cyclin D and E (*CCNE1*, *CCNE2*, *CDKN2A*, *E2F1*, and *RB1*), (ii) the prereplicative complex and DNA replication (*CDC45*, *CDC7*, *CDCA7*, *CDT1*, *MCM2*, and *ORC1*), (iii) the Fanconi anemia pathway (*CLSPN*, *FANCA*, *FANCE*, and *RAD54L*), and (iv) chromosome condensation (*CDC20*, *CDCA3*, *CENPA*, *DEPDC1*, *FAM83D*, and *SKA1*) (table S3; Materials and Methods: Enrichment analysis). Furthermore, most of the genes in the enriched clusters were found to be overexpressed on average in the RBNSig-high patients, suggesting that these clusters represent mRNA up-regulation (rather than loss of function) of both the canonical RB-regulated processes (i) and the noncanonical/indirectly RB-regulated processes (ii to iv) associated with chromosomal stability and mitotic fidelity. As expected, genes down-regulated in RBNSig-BC included key markers of cell cycle control, such as *RB1* and *CCND1*, but also genes from a diverse range of molecular processes including AP-1 transcription factor complex (*FOS* and *FOSB)*, neuronal system (*SLC18A2* and *SYT1*), hemostasis (*SERPINA5*, *F10*, *F13A1*, and *MMRN1*), and immunoregulatory signaling (*FCER1A*, *MS4A2*, and *CD22)* (table S3). Next, we assessed whether RBNSig-BC scores were confounded by the level of tumor immune infiltration (*27*). We did not find evidence of correlation between the immune infiltration and RBNSig-BC alignment scores (*P* = 0.46). As an additional control, we tested the gene expression patterns of RBNSig-BC in four independent datasets of retinoblastomas (*28*–*31*), which can spontaneously arise following the loss of *RB1* (*31*, *32*). All four datasets exhibited overexpression of genes that were up-regulated in RBNSig-BC confirming *RB1*-dependent expression of these genes (fig. S3B).

Since RB/E2F target genes are known correlates of poor outcome (*33*), we asked whether RBNSig-BC enables prioritization of aggressive tumors in TCGA BRCA cohort. To delineate aggressive RBness cancers independent of the bona fide *RB1* defects (patients with low Rb or pRb, *RB1* MTs, and/or deep deletions), we assessed the outcome of the RBNSig-high group’s patients with RBness (*n* = 328) independently. While the *RB1*-defective group confirmed an expected poor outcome [Hazard ratio (HR) = 2.2, 95% confidence interval (CI) = 1.05 to 4.64, *P* = 0.037; Fig. 2C], patients in the RBness group also had significantly worse outcome (independent of clinical covariates) when compared to the RBNSig-low group (HR = 1.56, 95% CI = 1.06 to 2.29, *P* = 0.023). Independent prognostic validation of RBNSig-BC in the Metabric breast cancer cohort (*34*) revealed similar association between the RBness group and poor outcome for both OS as well as disease-specific survival (DSS) (Fig. 2, D and E, and table S4). This association was maintained in estrogen receptor–positive (ER^+^) breast cancer subtype for both OS and DSS (fig. S3, C and D). However, *RB1*-defective and RBness groups in TNBC and HER2^+^ subtypes were not associated with significantly worse outcome compared to the RBNSig-BC–low group (fig. S3, E to H).

To elucidate molecular drivers of RBness other than *RB1* defects, we examined RBness cancers for the enrichment of mutations, amplifications, and deletions of known breast cancer driver genes (*35*). When compared to the *RB1*-defective group, patients in the RBness group showed significant enrichment for *PIK3CA* amplifications (OR = 6.54, *P* = 0.037; Fisher’s exact test) and, unexpectedly, a decrease in *PTEN* deletions, albeit driven by small numbers (OR = 0.2, *P* = 0.004; Fisher’s exact test) (Fig. 2F and table S5). Amplifications in either Cyclin D1 (*CCND1*) or Cyclin E1 (*CCNE1*), both of which are members of the RB pathway, were also enriched in the RBness group (OR = 2.10, *P* = 0.058; Fisher’s exact test). However, amplifications in *CCND1* were observed at similar rates in the RBNSig-low group. Further, amplifications in either *ESR1*, *FOXA1*, or *GATA3*, which are key members of the ER signaling pathway (*36*), showed increased prevalence in the RBness group compared to the *RB1*-defective group (OR = 2.24, *P* = 0.079; Fisher’s exact test). Collectively, amplifications in either *PIK3CA*, the RB pathway (*CCND1* or *CCNE1*), or ER signaling (*ESR1*, *FOXA1*, or *GATA3*) were found in 49.7% of patients with RBness, highlighting potential driver mechanisms that could explain RBness in the absence of *RB1* MTs and deletions. Overall, both the *RB1*-defective and RBness groups constituted predominantly aggressive subtypes of breast cancer [PAM50 (*37*): Basal-like and HER2-enriched] compared to the RBNSig-low group, which was composed of Luminal subtypes (Fig. 2F). This also suggests a limitation when interpreting the observed better outcome of patients in RBNSig-low group since most of these patients belong to Luminal-A subtype, which is known for its association with good prognosis (*37*).

### RBNSig-BC predicts response to chemotherapy and contemporary neoadjuvant treatments

**Fig. 3.**
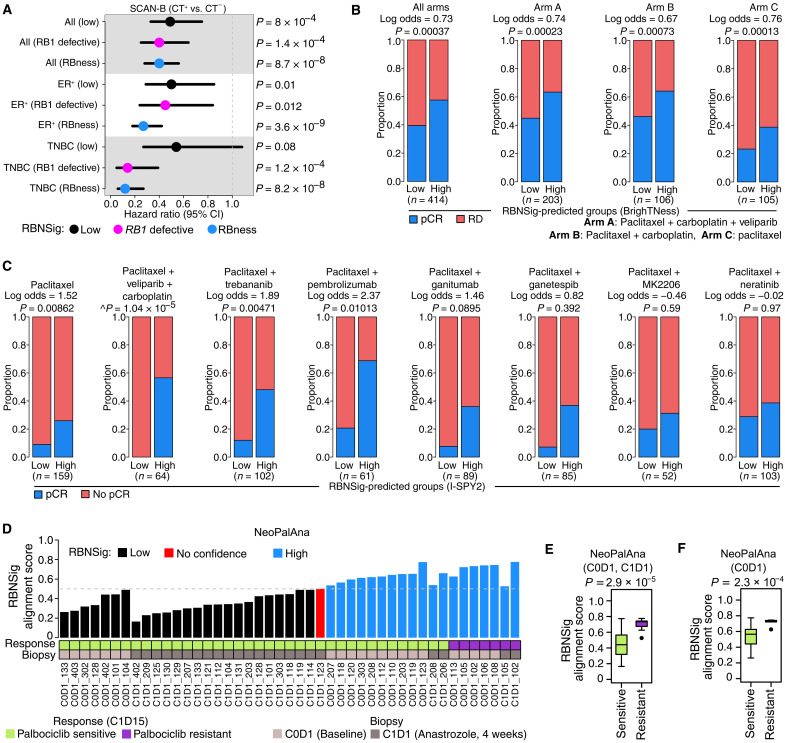
RBNSig of breast cancer subtypes. (**A**) Forest plot showing independent prognostic evaluation (OS) of TCGA-derived RBNSig-BC in the SCAN-B cohort (3250 patients) stratified by chemotherapy status. Survival analysis was performed separately on all patients (All), ER^+^ breast cancers, and TNBCs. (**B**) Predictive assessment of RBNSig-BC groups (low and high) in BrighTNess clinical trial (482 patients). RBNSig-BC–predicted risk groups were assessed against treatment response using logistic regression. RD, residual disease; pCR, pathologic complete response. “*n*” shows the combined number of patients in the RBNSig-BC–low and –high groups, excluding patients classified as “no confidence.” (**C**) Predictive assessment of RBNSig-BC groups (low and high) in eight treatment arms of I-SPY2 clinical trial (987 patients) using logistic regression (adjusted for HR status). no pCR, did not achieve pCR. *n* shows the combined number of patients in the RBNSig-BC–low and –high groups, excluding patients classified as “no confidence.” “^” indicates absence of log odds and *P* value from logistic regression model, and instead *P* value from Fisher’s exact is reported since no patients with pCR were present in the RBNSig-BC–low group. (**D**) Bar plot showing alignment scores of samples in the NeoPalAna breast cancer trial. Horizontal dashed gray line marks the classification point (50% signature alignment). All samples included in this analysis were anastrozole treated (*n* = 45). Matched gene expression profiles at baseline (C0D1), anastrozole treatment (C1D1) and primary end-point (C1D15), and Ki67 measurements (after anastrozole + palbociclib treatment) for all samples were not available in the original study. (**E** and **F**) Box plots showing distribution of RBNSig-BC–predicted alignment scores for all C0D1 and C1D1 samples (E) and C0D1 samples (F) in the NeoPalAna trial grouped by treatment response. Statistical comparison was performed using the Welch’s *t* test.

Although RBNSig-BC was able to identify aggressive tumors with poor outcome in relatively old retrospective clinical cohorts such as TCGA and Metabric, we also acknowledge the established association between *RB1* defects and sensitivity to chemotherapy (*38*–*41*). Therefore, we asked whether RBness cancers respond to recent chemotherapy agents in a contemporary breast cancer cohort: SCAN-B (*n* = 3250). Using the TCGA-derived RBNSig-BC, SCAN-B patients were classified into high, low, and no confidence groups (table S6). *RB1*-defective patients were enriched in the RBNSig-BC–high group (OR = 6.1, *P* < 2.2 × 10^−16^; Fisher’s exact test), confirming the reproducibility of RBNSig-BC in this contemporary cohort. To test association with chemotherapy, we divided the RBNSig-BC–high group into bona fide *RB1*-defective cancers and RBness cancers. Chemotherapy-treated patients in both groups showed better OS compared to the chemotherapy-naïve patients in these group (*RB1* defective: HR = 0.40, 95% CI = 0.25 to 0.64, *P* = 1.4 × 10^−4^; RBness: HR = 0.40, 95% CI = 0.28 to 0.56, *P* = 8.7× 10^−8^; Fig. 3A and fig. S4). This association remained statistically significant when SCAN-B patients were further stratified into ER^+^ and TNBC subtypes (ER^+^ *RB1* defective: *P* = 0.012; ER^+^ RBness: *P* = 3.6 × 10^−9^; TNBC *RB1* defective: *P* = 1.2 × 10^−4^; TNBC RBness: *P* = 8.2 × 10^−8^; Fig. 3A and fig. S4). These data suggest that some of the patients with RBness exhibiting phenocopy of *RB1*-defective cancers could potentially benefit from chemotherapy.

We further challenged the observed association between RBNSig-BC and chemotherapy response in a recent TNBC phase 3 neoadjuvant chemotherapy trial “BrighTNess” (*n* = 482) (*42*). Across the entire dataset irrespective of the treatment arm (Arm A: paclitaxel plus carboplatin plus veliparib, Arm B: paclitaxel plus carboplatin plus veliparib placebo, and Arm C: paclitaxel plus carboplatin placebo plus veliparib placebo), patients with pathologic complete response (pCR; defined as the disappearance of all invasive cancer in the breast after completion of neoadjuvant chemotherapy) were enriched in the RBNSig-BC–high group (log odds = 0.73, *P* = 3.7 × 10^−4^; Fig. 3B and table S7), suggesting that patients who are *RB1* defective and patients with RBness are likely sensitive to chemotherapy agents such as paclitaxel or carboplatin in neoadjuvant setting. This association remained statistically significant when each of the three treatment arms were analyzed independently, providing evidence that RBNSig-BC can further improve patient selection for neoadjuvant paclitaxel combination with carboplatin (Fig. 3B, Arms A to C). Given that the TNBC neoadjuvant standard-of-care treatment options are rapidly evolving, with recent approval for combination of immunotherapy with carboplatin, taxane, and anthracycline, we further tested the suitability of RBNSig-BC in contemporary neoadjuvant drug combinations that are currently investigated for breast cancer as a part of I-SPY2 adaptive phase 2 neoadjuvant trial (n = 987) (*43*). I-SPY2 assessed 10 drug combinations with paclitaxel targeting different aspects of tumor biology. Of these, we focused on eight drug combinations, excluding treatment arms with approved HER2-directed agents (pertuzumab and trastuzumab) since effective targeted therapies for the latter groups already exist (*44*). The RBNSig-BC–high group showed higher pCR rates compared to the RBNSig-BC–low group in the paclitaxel arm (log odds = 1.52, *P* = 0.0086; Fig. 3C). Consistent with our results in the BrighTNess trial, the RBNSig-BC–high group also showed significant enrichment of patients with pCR compared to the RBNSig-BC–low group when carboplatin and veliparib were added to paclitaxel (*P* = 1.04 × 10^−5^). Similar enrichment was observed in combination treatment arms of trebananib (antiangiogenic peptide inhibitor), pembrolizumab (anti–PD-1 immunotherapy), and ganitumab (*IGF1R* inhibitor) (Fig. 3C). While the RBNSig-BC–high group contained a higher fraction of patients with pCR compared to the RBNSig-BC–low group in other treatment arms, this increased fraction was not significant. These data further highlight potential translational application of RBNSig-BC as a biomarker to assist in patient selection to further improve pCR rates in contemporary neoadjuvant treatment setting.

### RBNSig-BC predicts response to CDK4/6 inhibitors

Recent studies have shown *RB1* inactivation as one of the potential mechanisms underlying resistance to CDK4/6 inhibitors in ER^+^ breast tumors (*10*–*12*). Hence, we asked whether RBNSig-BC could be used to identify patients harboring early transcriptional indicators of RBness and therefore unsuitable for CDK4/6 inhibitors. To test this, we applied RBNSig-BC to gene expression profiles of ER^+^ breast cancer patients in a phase 2 neoadjuvant trial “NeoPalAna” (*45*). Briefly, NeoPalAna evaluated the efficacy of palbociclib (a selective inhibitor of CDK4 and CDK6) in combination with anastrozole in ER^+^/HER2^−^ patients who fail to respond to anastrozole monotherapy for 4 weeks (C1D1). The primary end point of the study was complete cell cycle arrest (Ki67 ≤ 2.7%) at 2 weeks of combination therapy (C1D15) following C1D1. We applied RBNSig-BC to palbociclib-naïve tumor biopsies taken at the baseline (C0D1) and anastrozole monotherapy–treated samples (C1D1) (Fig. 3D). Overall, RBNSig-BC–predicted alignment scores for palbociclib-resistant samples were significantly higher compared to the palbociclib-sensitive samples (C0D1 + C1D1 samples: *P* = 2.9 × 10^−5^; C0D1 samples: *P* = 2.3 × 10^−4^; Fig. 3, E and F). Specifically, RBNSig-BC classified all seven palbociclib-resistant samples (five patients; five C0D1 and two C1D1 biopsies) as RBNSig-high group with 100% sensitivity. Since only one of these five palbociclib-resistant patients had an *RB1* mutation (patient 106), the presence of RBness phenocopy in the other four patients was potentially a biomarker of palbociclib resistance. RBNSig-BC also classified an additional 12 of 38 palbociclib-sensitive samples as RBNSig-high (specificity = 68.42%). Of the palbociclib-sensitive group, two patients carried *RB1* mutations (MT) (patient identifiers: 131 and 203) at the C0D1 biopsy. For patient 131, gene expression profile at C0D1 was not available; however, its C1D1 biopsy was correctly classified as RBNSig-low. For patient 203, RBNSig-BC classified the *RB1*^MT^ C0D1 biopsy as RBNSig-high, while *RB1*^WT^ C1D1 biopsy was correctly classified as RBNSig-low. This change in the classification of RBNSig-BC for patient 203 correctly mirrors the change in subclonal composition of the tumor that lost the *RB1*^MT^ subclone (variant allele frequency at C0D1 = 5.4%) over the course of neoadjuvant treatment. This highlights the importance of dynamic profiling of tumors as they evolve through treatment for better clinical decision-making.

### Benchmarking of RB-related signatures reveal limited overlap between genes and superior performance of RBNSig-BC

**Fig. 4.**
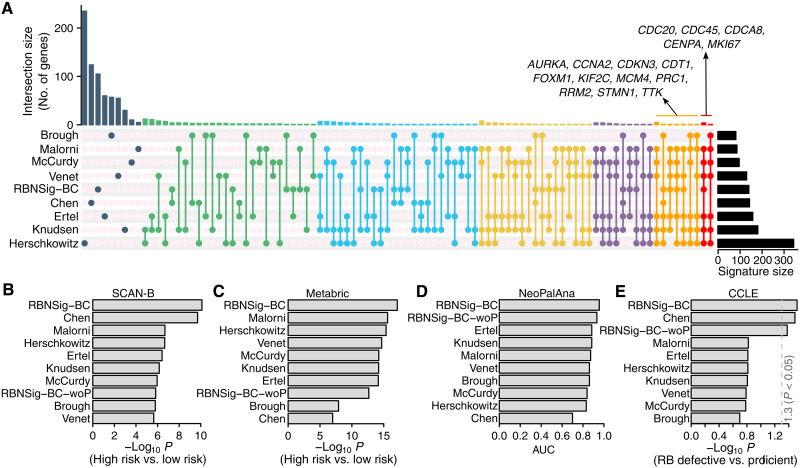
Benchmarking of RBNSig-BC. (**A**) Upset plot showing the overlap between RBNSig-BC and other RB-related gene signatures. (**B**) Prognostic performance comparison of RB-related gene signatures in SCAN-B breast cancer cohort. For each signature, per-patient risk score was estimated using Singular value decomposition (Materials and Methods: Benchmarking signatures). Risk scores were trichotomized using tertiles, and low and high groups were compared using the Cox proportional hazards model. Wald-test *P* value was used to assess the signature’s performance. (**C**) Prognostic performance comparison of RB-related gene signatures in the Metabric breast cancer cohort. (**D**) Predictive performance comparison of RB*-*related gene signatures in the NeoPalAna clinical trial. Per-patient risk score was calculated as specified in (B) and association with treatment response (sensitive or resistant) was evaluated using area under the receiver operating characteristic curve (AUC). (**E**) Performance comparison of RB*-*related gene signatures in breast cancer cell lines. Per-cell line signature score was calculated as specified in (B), and comparison between the *RB1*-defective and *RB1*-proficient cell lines was performed using the Welch’s *t* test. RBNSig-BC-woP represents a differential gene expression–based signature derived in the same dataset (TCGA BRCA, *n* = 81) that was used for the development of RBNSig-BC however without taking protein data into consideration.

To compare the constituent genes and benchmark performance of RBNSig-BC with previously identified signatures of RB loss, we collated a panel of eight RB-related signatures constituting five RB loss signatures of breast cancer (*15*, *18*–*20*, *39*), a proliferation signature (*46*), a pan-cancer *RB1* loss signature (*17*), and a *CDK2* activity signature (*47*). Consistent with previous findings (*17*, *18*, *39*), these signatures showed limited overlap of genes (Fig. 4A). Only five genes were shared by at least seven signatures, encompassing genes involved in cell cycle, proliferation, and DNA replication during mitosis (*CDC20*, *CDC45*, *CDCA8*, *CENPA*, and *MKI67*). An additional 11 genes were shared by six signatures (Fig. 4A). Despite this limited overlap, these signatures shared known pathways implicated in *RB1*-defective cancers such as cell cycle, DNA replication and synthesis, and DNA repair (fig. S5 and table S8), suggesting higher-order similarities in the underlying biology of these signatures. Of these signatures, Knudsen *et al.* (*20*) and Malorni *et al.* (*19*), both of which were designed for predicting palbociclib resistance, shared 17 genes each with RBNSig-BC including 11 genes (*KIF2C*, *RAD54L*, *KIFC1*, *ORC1*, *CDCA8*, *CDC45*, *FAM83D*, *CDC20*, *CENPA*, *RRM2*, and *PIF1*) that were present in all three signatures. This overlap is a likely explanation for the predictive performance of RBNSig-BC in the NeoPalAna trial (Fig. 3, D to F).

Next, we systematically compared the performance of RBNSig-BC against these eight signatures and a control signature developed using the same approach as RBNSig-BC without proteomics data (RBNSig-BC-woP). A coherent signature scoring and benchmarking approach was implemented using two breast cancer clinical cohorts (SCAN-B and Metabric), one breast cancer clinical trial (NeoPalAna), and breast cancer cell lines (CCLE) with known *RB1* status (Materials and Methods: Benchmarking RBNSig-BC). In the breast cancer clinical cohorts, while all signatures were significantly associated with poor outcome, RBNSig-BC was superior to all other signatures in accurate identification of low- and high-risk patients (Fig. 4, B and C). In the NeoPalAna trial, RBNSig-BC’s classification was the strongest predictor of CDK4/6i response compared to other signatures (Fig. 4D). When tested in breast cancer cell lines, RBNSig-BC, Chen *et al.* (*17*) and RBNSig-BC-woP were the only signatures predictive of known *RB1* defects (Fig. 4E).

### Cancer type–specific RBNSigs highlight RBness as a general pan-cancer phenomenon

**Fig. 5.**
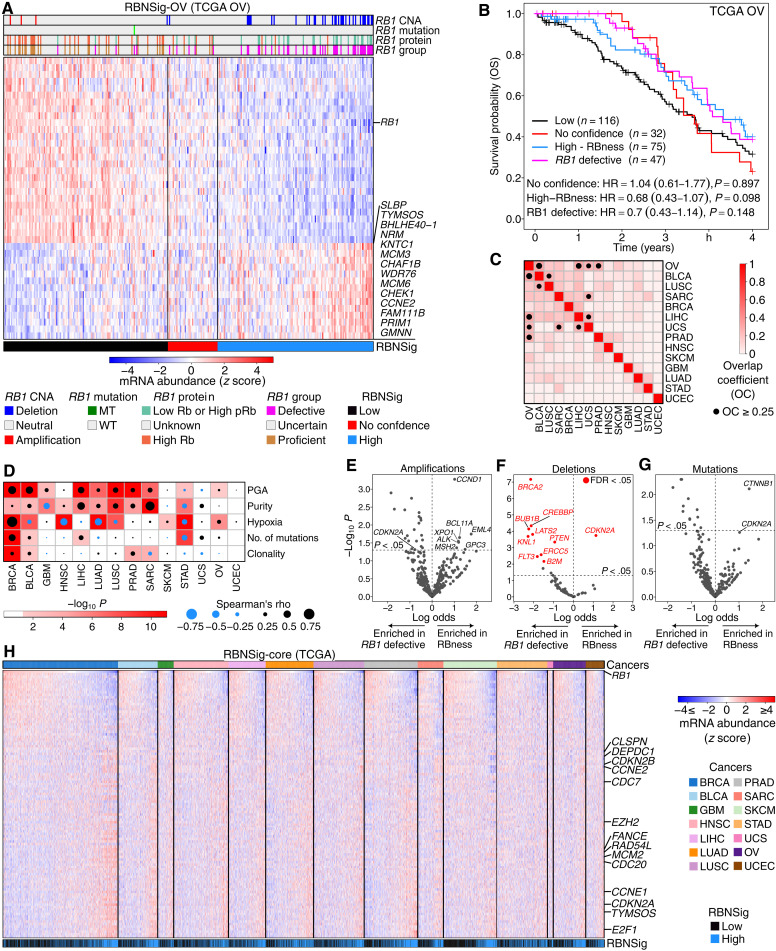
RBNSig of high-grade ovarian cancer and pan-cancer. (**A**) Heatmap showing the RBNSig of ovarian cancer in TCGA’s high-grade serous ovarian adenocarcinoma (OV) dataset (246 patients). Vertical black lines in the heatmap show patient stratification into three RBNSig-OV groups: high (>55% signature alignment; RBNSig, blue), low (<45% signature alignment; RBNSig, black), and no confidence (45 to 55% signature alignment; RBNSig, red). *RB1* CNA indicates copy number states: amplification, neutral, and deep deletion. *RB1* mutation indicates mutation status: MT, truncating mutation; WT, nontruncating mutation; or wild type. *RB1* protein indicates low Rb abundance or high phosphorylated Rb (pRb) relative to total Rb. *RB1* status indicates *RB1*-defective and *RB1*-proficient status inferred from CNA, mutation, or proteomic profiles. (**B**) Prognostic assessment of the RBNSig-OV in TCGA’s chemotherapy-treated cohort. The RBNSig-low group was treated as the reference group in a multivariable Cox proportional hazards model adjusted for age, tumor size (T-stage), and grade. (**C**) Heatmap showing OC between the RBNSig of different cancer types. OC ≥ 0.25 is highlighted with a black circle with its size relative to the magnitude of OC. (**D**) Heatmap showing correlation between cancer type–specific RBNSig and markers of tumor aggressivity. Three white cells for UCEC without a circle indicate lack of observations (*n* < 5) to test correlation. (**E** to **G**). Pan-cancer candidate driver gene enrichment in *RB1*-defective and RBness groups. (**H**) Heatmap showing mRNA abundance profile (*z* scores) of the pan-cancer RBNSig-core, that was defined as the set of genes present in at least 3 (20%) of 14 cancer types. Columns (samples) in each cancer type were sorted by *RB1* mRNA abundance. Rows (genes) were sorted on the basis of pan-cancer correlation between *RB1* and each gene. For clarity, samples predicted as no confidence are not shown.

To test the generalization of our proteogenomic approach to other cancer types, we repeated the discovery of the RBNSig in a discovery subset (*n* = 89) of TCGA’s high-grade serous ovarian adenocarcinoma (OV) (*48*) and tested it in the entire TCGA OV cohort (*n* = 304). The resulting signature (RBNSig-OV) was composed of 41 genes (Fig. 5A, fig. S6, and table S9) and successfully validated in an independent proteogenomic cohort (CPTAC 2/3, *n* = 81; fig. S7) where the predicted RBNSig-OV–high group was enriched with samples harboring either an *RB1* deep deletion, an *RB1* MT, low Rb abundance, or high pRb (*n* = 35, OR = 88.57, *P* = 3.71 × 10^−10^, Fisher’s exact test). While the constituent genes in RBNSig-OV showed a limited overlap of four genes (*RB1*, *CCNE2*, *FAM111B*, and *TYMSOS*) with the RBNSig of breast cancer (RBNSig-BC), the RBNSig-BC genes showed similar expression profile in ovarian cancer albeit with smaller effect size, suggesting similarities in the biology of *RB1*-defective cancers of breast and ovary (fig. S8).

The standard of care for high-grade serous OVs includes surgery followed by platinum-taxane chemotherapy (*48*). However, response to these agents remains heterogeneous (*49*), and, hence, we asked whether RBNSig-OV is associated with chemotherapy response. Among the RBNSig-OV–high group, both *RB1*-defective and RBness subgroups showed a trend of better OS compared to the RBNSig-low group indicating increased chemosensitivity, but this was not statistically significant (*RB1* defective: HR = 0.7, 95% CI = 0.43 to 1.14, *P* = 0.148; RBness: HR = 0.68, 95% CI = 0.43 to 1.07, *P* = 0.098; Fig. 5B).

Inactivation of *RB1* was recognized in early studies to be a recurrent hallmark of tumor development and progression across many cancer types (*50*, *51*). Therefore, we applied the RBNSig discovery pipeline to the transcriptomes of three further cancer types where matched proteogenomic profiles were available [CPTAC 2/3: lung adenocarcinoma (LUAD), glioblastoma (GBM), endometrial carcinoma (UCEC)] and an additional 27 cancer types where only genomic profiles were available (Materials and Methods: Pan-cancer identification of RBNSigs). Of these cancer types, 12 successfully generated a valid RBNSig (tables S10 and S11). The number of genes in RBNSig across different cancer types varied considerably, with prostate adenocarcinoma (PRAD) having the largest signature (491 genes) and uterine carcinosarcoma (UCS) the smallest signature (5 genes) (table S10). The overlap between the constituent genes of RBNSig for different cancer types remained low to moderate, with OV showing the highest overlap coefficient (OC) with BLCA (OC = 0.32) and liver carcinoma (LIHC, OC = 0.30) (Fig. 5C). To investigate whether the genes in RBNSig of different cancers were predictive of aggressive disease as observed for the RBNSig of breast cancer and OV, we examined the relationship between RBNSig alignment scores (Materials and Methods: Alignment score and signature direction) and key markers for disease aggression including percent genome alteration (PGA), tumor purity, hypoxia, mutation burden, and clonal heterogeneity (*52*–*55*). Except for stomach adenocarcinoma (STAD) and uterine cancers (UCS and UCEC), all other cancers showed significant positive correlation with at least one of these markers of tumor aggressivity (Spearman’s correlation *P* < 0.05; Fig. 5D). The number of mutations in STAD showed significant negative correlation with RBNSig alignment scores, which is consistent with previous findings describing high tumor mutation burden as a marker of good prognosis in gastric cancers (*56*, *57*). These data further support RBness as a pan-cancer phenomenon associated with tumor aggression.

To identify pan-cancer molecular drivers in patients exhibiting RBness in the absence of known *RB1* defects, we interrogated TCGA molecular datasets to test for enrichment of somatic mutations, amplifications, and deletions using high-confidence cancer driver genes (table S12; Materials and Methods: Association with candidate driver alterations) (*58*). Amplifications in *CCND1*, *BCL11A*, *EML4*, *XPO1*, *ALK*, *MSH2*, and *GPC3* were enriched in RBness cancers compared to *RB1*-defective cancers (*P* < 0.05; Fig. 5E). Deletions in cyclin-dependent kinase inhibitor 2A (*CDKN2A*/p16) and mutations in *CDKN2A* and β-catenin (*CTNNB1*) were also enriched in the RBness group (Fig. 5, F and G). While *CDKN2A* deletions were enriched in RBness cancers, conversely, amplifications in *CDKN2A* were enriched in *RB1*-defective cancers. These data provide basis for understanding the differences in molecular drivers of RBness compared to *RB1*-defective tumors that still result in similar transcriptional phenotypes.

To reconcile differences in RBNSig of 14 cancers, we hypothesized that a core universal functional module may exist across cancers and that this could be captured by a higher-order analysis of RBNSig of different cancers. To test this hypothesis, we first defined a core set of pan-cancer RBNSig genes that were present in at least three (20%) of the 14 cancer types. The resulting pan-cancer signature, called RBNSig-core, was composed of 107 genes (Fig. 5H and table S13). Up-regulated genes in RBNSig-core were concordant with *RB1*-dependent up-regulation of key genes observed in retinoblastomas (fig. S9A). To characterize the shared pan-cancer biology captured by RBNSig-core, we performed STRING enrichment analysis, which revealed two primary modules: (i) processes and complexes playing a role in DNA replication, Fanconi anemia pathway, the prereplicative complex and the origin of replication complex and (ii) processes and complexes playing a role during the G_1_ cell cycle phase. Both modules mediate chromosomal integrity during mitosis (fig. S9B and table S14). These data suggest that while differences in RBNSig of different cancer types exist, they also converge on well-known shared biology of *RB1*-defective cancers.

Next, we evaluated the prognostic and predictive performance of RBNSig-core in 14 cancer types where *RB1* defects were frequently observed (table S10). RBNSig-core was prognostic in breast, liver, stomach, skin, and lung cancer datasets and was also predictive of CDK4/6i response in breast cancer (fig. S9C). RBNSig-core’s prognostic performance remained superior to two previously published pan-cancer signatures of *RB1* loss (*17*) and *CDK2* activity (*47*) but inferior to the performance of RBNSig-BC overall (fig. S9, D to G). While it was not possible to generate valid RBNSigs for 18 other cancer types that lacked frequent *RB1* defects or have limited proteogenomic data (table S10), we asked whether there exist patients with RBness in these additional cancer types. In the absence of *RB1* defects as a ground truth for these cancers, we assumed that patients with RBness are more likely to exhibit poor clinical outcome than those without RBness. To test this phenomenon, we applied RBNSig-core to patients in these 18 cancer types and correlated the resulting RBNSig-core–predicted high and low groups with patients’ OS. The RBNSig-core–predicted high group was associated with poor outcome in five cancer types (fig. S9H), suggesting the presence of RBness in cancers that lack classical *RB1* genetic defects.

### RBness phenocopies therapeutic vulnerabilities and drug response of *RB1*-defective cancers

**Fig. 6.**
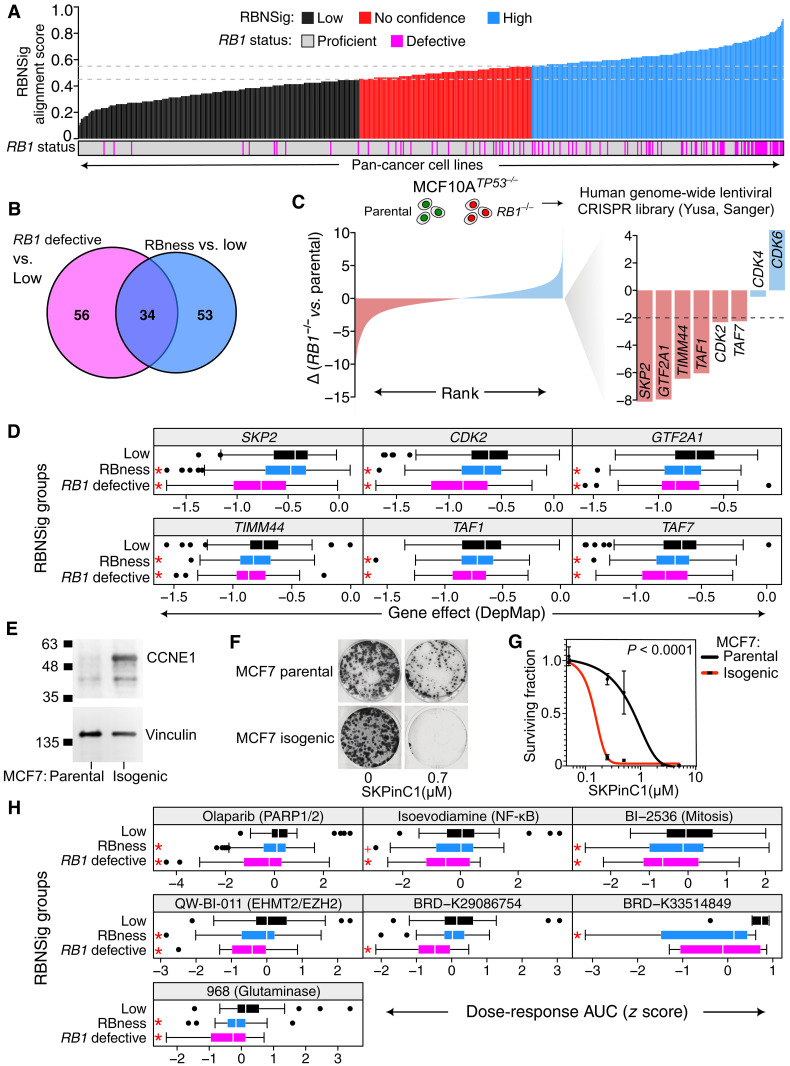
Using RBNSig-core to define candidate synthetic vulnerabilities and in vitro validation. (**A**) Bar plot showing the classification of DepMap cancer cell lines by RBNSig-BC into low and high groups, with *RB1*-defective lines highlighted in purple. (**B**) Venn diagram of overlap between candidate synthetic lethal genes identified by comparing gene effect profiles between *RB1*-defective versus RBNSig-low and RBness versus RBNSig-low groups. (**C**) CRISPR-Cas9 screen using the isogenic MCF10A*^TP53−/−^* breast epithelial cell line pair with and without *RB1*^−/−^. Ranked bar plot showing the difference in the viability *z* scores (highest, left to lowest, right). In zoomed bar plot, key *RB1* synthetic lethal genes are highlighted in pink. Key *RB1* resistance–associated genes are highlighted in blue. Dashed line (Δ = −2) indicates a minimum acceptable difference deemed appropriate to be considered a candidate vulnerability. (**D**) DepMap CRISPR-Cas9 gene effect profiles of exemplar genes that were synthetic lethal with both *RB1* defects and RBness and validated using the isogenic *RB1* MCF10A^*TP53*−/−^ CRISPR-Cas9 screen when *RB1*-defective or RBness group was compared to the RBNSig-low group (*P < 0.05, one-sided Welch’s *t* test). (**E**) CCNE1 expression determined by Western blot of lysates derived from MCF7 parental and isogenic MCF7^*CCNE1*amplified^ cells. Vinculin was probed as a loading control. (**F**) Representative images of parental and isogenic MCF7 cells treated with or without 0.7 μM of SKP2 inhibitor (SKPinC1) for 2 weeks. (**G**) Dose response curves of parental and isogenic MCF7 cells when exposed to increasing doses of SKPinC1. Two-way analysis of variance (ANOVA) was used to assess significance of difference between the curves. (**H**) CTD^2^ dose-response AUCs (*z* scores) of compounds that showed selective sensitivity in *RB1*-defective or RBness groups when compared to cell lines in the RBNSig-low group. Compound name and their target gene/biological process (where available) are shown in parenthesis (*P < 0.05, +*P* = 0.0548, one-sided Welch’s *t* test).

Because RBness shared the transcriptional profile of *RB1*-defective cancers and was associated with aggressive forms of cancer, we next asked whether these cancers shared therapeutic vulnerabilities with *RB1*-defective cancers that could be exploited for designing targeted therapies. To answer this question, we first applied the patient-derived RBNSig-core to a lineage-matched pan-cancer mRNA dataset of cell line models (*n* = 563) and classified the cell lines as RBNSig-low, no confidence, or -high (table S15). *RB1*-defective cell lines were enriched in the RBNSig-high group (69 of 100, OR = 11.12, *P* = 3.66 × 10^−16^, Fisher’s exact test; Fig. 6A) confirming validity of the patient-derived RBNSig-core signature in cell line models. Second, using these RBNSig-core–predicted groups, we interrogated publicly available genome-wide CRISPR-Cas9 screens carried out in these same cell lines (DepMap) (*59*, *60*) to identify vulnerabilities associated with RB pathway defects. Similar to patient analyses described earlier in this study, known *RB1*-defective cell lines were evaluated independent of RBness group. To do this, we performed two comparisons: (i) *RB1*-defective versus RBNSig-low and 2) RBness versus RBNSig-low, to define candidate genes that were synthetic lethal with *RB1* defects and RBness. Comparison of DepMap CRISPR-Cas9 gene effect profiles revealed 90 and 87 candidate genes that exhibited synthetic lethality with *RB1* defects and RBness, respectively (*P* < 0.05, one-sided Welch’s *t* test; Fig. 6B and tables S16 and S17; Materials and Methods: CRISPR perturbation screens analysis). Of these, 34 genes were shared synthetic lethal vulnerabilities between the *RB1*-defective and RBness groups (a statistically significant overlap, *P* = 2.08 × 10^−57^, Fisher’s exact test), suggesting that these genes are likely to phenocopy treatment response to *RB1* targeting agents in the RBness group. In addition, there were 56 and 53 candidate synthetic lethal genes exclusive to *RB1*-defective and RBness groups, respectively. To account for differences in cell line histology, we repeated this analysis using the generalized linear models adjusted for histotype, where we also found a significant overlap in synthetic lethal genes of *RB1*-defective and RBness groups (*P* = 3.07 × 10^−14^, Fisher’s exact test; fig. S10A).

To validate these results in vitro, we analyzed a genome-wide CRISPR-Cas9 loss of function screen using an isogenic MCF10A^*TP53*−/−^ breast epithelial cell line pair with or without *RB1* deletion (Fig. 6C). The RNA interference knockdown of key selective gene dependencies of *RB1* (*SKP2*, *E2F3*, and *TFDP1*) in MCF10A^*TP53*−/−*RB1*−/−^ cells corroborated our findings made using the DepMap dataset and confirmed the suitability of this model when interpreting *RB1*-associated gene dependencies (fig. S10, B to D). Furthermore, RNA sequencing (RNA-seq) profiling of MCF10A^*TP53*−/−^ and MCF10A^*TP53*−/−*RB1*−/−^ cells also confirmed *RB1*-dependent activity of RBNSig-core genes (fig. S10E). By comparing CRISPR-Cas9 screen data in MCF10A^*TP53*−/−*RB1*−/−^ versus MCF10A^*TP53*−/−^ cells, we identified 764 candidate *RB1* synthetic lethal genes. These included known vulnerabilities of *RB1*-defective cells such as *SKP2*, *CDK2*, and *TAF1* (*15*). Among these 764 genes, there was a strong enrichment of RBNSig-core–identified candidate synthetic lethal genes from DepMap dataset (47 of 143; *P* = 1.16 × 10^−28^, Fisher’s exact test). Thirteen of 34 synthetic lethal genes common between RBness and *RB1*-defective cell lines successfully validated in the MCF10A^*TP53*−/−*RB1*−/−^ screen, including *SKP2*, *CDK2*, *GTF2A1*, *TIMM44*, *TAF1*, and *TAF7* (Fig. 6D and fig. S10F). These data further support the notion that RBness phenocopies genetic vulnerabilities of *RB1*-defective cancers and hence opens the possibility of repurposing treatment strategies for patients exhibiting RBness in the absence of *RB1* genomic defects. Of the vulnerabilities identified as exclusive to *RB1*-defective or RBness-harboring cell lines, a further 28 and 6 genes respectively were validated in the MCF10A^*TP53*−/−*RB1*−/−^ screen (figs. S11 and S12), suggesting potentially distinct synthetic lethal candidates present in these two subgroups of RBNSig-high cell lines. Of the validated vulnerabilities of RBness, poly(A)-binding protein 1 (*PABPC1*) and mitochondrial dynamin–like GTPase (optic atrophy 1, *OPA1*) showed the strongest effect (fig. S12). Both *PABPC1* and *OPA1* are aberrantly expressed in many cancers and are known to promote tumorigenesis and progression, thereby serving as promising candidates to target in RBness cancers (*61*, *62*). To benchmark RBNSig-core’s ability in detecting synthetic lethal dependencies, we compared its performance with eight other previously published RB-related gene signatures (*15*, *17*–*20*, *39*, *46*, *47*). For this analysis, a synthetic lethal gene list identified from the isogenic *RB1* MCF10A*^TP53−/−^* CRISPR-Cas9 screen was considered as the ground truth. Next, we tested the overlap of this gene list with the synthetic gene lists identified by each of the RB-related gene signatures using the DepMap dataset. RBNSig-core was superior in performance compared to other signatures in predicting the candidate synthetic lethal genes identified in the isogenic *RB1* MCF10A*^TP53−/−^* CRISPR-Cas9 screen (fig. S13A).

While the isogenic *RB1* MCF10A*^TP53−/−^* cells were ideal for establishing the causal relationship between *RB1* loss and corresponding synthetic lethal genes, we acknowledged that these cells may not mirror those cancers that exhibit RBness where it occurs in the absence of genomic *RB1* defects. As shown earlier (Fig. 2F), RBness breast cancers frequently exhibited cyclin E1 (*CCNE1*) amplifications compared to *RB1*-defective breast cancers. We therefore used a previously described isogenic MCF7 *CCNE1*–amplified cell line model of RBness (MCF7 and a daughter clone with *CCNE1* amplification, MCF7^*CCNE1*amplified^) (*11*, *63*) (Fig. 6E). Gene expression data from isogenic cells confirmed differential activity of RBNSig-core signature genes between the parental MCF7 and isogenic MCF7^*CCNE1*amplified^ daughter clone (fig. S13B). To assess whether this isogenic RBness model phenocopies synthetic lethal effects associated with *RB1* defects, we evaluated the sensitivity of isogenic RBness cells (MCF7^*CCNE1*amplified^) to a toolbox *SKP2* small-molecule inhibitor SKPinC1. This inhibitor has previously been shown to elicit synthetic lethality in isogenic *RB1* MCF10A^*TP53*−/−^ cells (*15*). We found that MCF7^*CCNE1*amplified^ cells were more sensitive to *SKP2* inhibition when compared to parental MCF7 cells (Fig. 6, F and G), corroborating the hypothesis that RBness cancers harbor synthetic lethal targeting opportunities similar to those found in *RB1*-defective cancers.

To further test the translational potential of RBNSig, we interrogated drug response data from 461 pan-cancer cell lines when exposed to 481 compounds from the Cancer Target Discovery and Development (CTD^2^) project (*64*, *65*). Consistent with the previous analyses, we compared the dose-response scores between (i) *RB1*-defective versus RBNSig-low and (ii) RBness versus RBNSig-low, to identify candidate drugs that show sensitivity in *RB1*-defective and RBness cell lines. These results highlighted five drugs that were significantly associated with reduced viability in both *RB1*-defective and RBness cell lines, while one drug was exclusively associated with reduced viability in each group (Fig. 6H and table S18). Five drugs that showed reduced viability in both *RB1*-defective and RBness cell lines included compounds of clinically actionable targets such as PARP1/2, NF-κB pathway, mitosis, EHMT2/EZH2, and metabolic enzyme glutaminase (GLS1). When compared to other RB-related signatures (*15*, *17*–*20*, *39*, *46*, *47*), RBNSig-core was a superior predictor in identifying these drug associations (fig. S13C). In summary, these data further underscore the hypothesis that RBness cancers phenocopy molecular and clinical characteristics of *RB1*-defective cancers and therefore could benefit from the same treatment strategies.

## DISCUSSION

This work introduces a transcriptional phenocopy of *RB1*-defective cancers, called RBness, that identifies aggressive forms of cancers. These aggressive cancers mimic the altered expression patterns observed in tumors with structural genomic defects in the *RB1* gene such as MTs and homozygous loss, which are frequently observed in human cancers (*50*). To capture RBness phenocopy, a proteogenomic signature extraction methodology was designed and applied to a series of clinical cohorts and trials. The integration of mass-spectrometry protein data, along with mutational, copy number, and mRNA abundance data, were shown to be a powerful tool in capturing not only genomic and transcriptomic alterations but also posttranslational modifications. The functional complexity of the Rb protein, resulting from varied multisite phosphorylation, needs further investigation in preclinical and clinical studies aiming at quantifying Rb functional states and their role in human cancers. Here, Rb protein abundance data played a key role in the robust identification of aggressive breast (ER^+^ and TNBC) and ovarian tumors that are sensitive to chemotherapy, therefore supporting the use of high-throughput proteomic data in biomarker discovery.

Previous studies (*38*–*41*) have highlighted significant association between *RB1* defects and improved chemotherapy outcome. Our data corroborate this association in contemporary cohorts of breast cancer and OV for both *RB1*-defective patients and additional patients who exhibit RBness. These patients could benefit from similar treatments to *RB1*-defective patients, such as combination therapies including chemotherapy and immunotherapy, especially in the neoadjuvant setting. This is evidenced by our results in the TNBC neoadjuvant clinical trial BrighTNess (*42*) and breast cancer neoadjuvant trial I-SPY2 (*43*), showing significantly higher pathological complete response rate in RBNSig-BC–high cancers. While the chemotherapy benefit for *RB1*-defective or Rbness cancers is a key finding and may be directly applicable to some cancer types, the emerging landscape of TNBC neoadjuvant standard-of-care treatment options that now includes a combination of immunotherapy with carboplatin, taxane, and anthracycline, adds to the complexity of using RBNSig-BC for TNBC (*66*). Therefore, the translational application of RBNSig-BC in TNBC neoadjuvant setting would require further testing in appropriately sized patient studies with contemporary treatment arms.

Selection of genomic amplifications is a hallmark of tumorigenesis (*67*, *68*) and often represent cancer cell vulnerabilities (*69*). While our data suggested several pan-cancer genomic gains/amplifications specific to patients with RBness patients (*CCND1*, *BCL11A*, *EML4*, *XPO1*, *ALK*, *MSH2*, and *GPC3*), none were a selective gene dependency in cancer cell lines, suggesting a potentially redundant contribution in driving RBness. In breast cancer alone, up to 50% of patients with Rbness in the absence of *RB1* genomic alterations harbored amplifications in *PIK3CA*, RB (*CCND1* and *CCNE1*), and ER signaling modules (*ESR1*, *FOXA1*, and *GATA3*). This offers exciting therapeutic opportunities such as the targeting of the PI3K pathway (*70*) for patients exhibiting RBness. Further, *CCND1*, a transcriptional target of ER signaling, along with its binding partners CDK4/6, modulates phosphorylation of Rb (*71*, *72*). Hence, palbociclib (*73*), a selective CDK4/6 inhibitor, for CDK4/6-driven *RB1* defects is also a promising option for RBNSig-BC–identified aggressive cancers. However, data shown in this study also highlight RBNSig-BC as a potential biomarker of palbociclib resistance in a small phase 2 clinical trial NeoPalAna (*45*). Although these data should be treated with caution because of small sample size, assessment of palbociclib response only after a 2-week treatment window and lack of Rb protein and phosphorylation status; a possible interpretation is that the patients exhibiting transcriptional phenocopy of *RB1* defects especially in the absence of CDK4/6-driven phospho-Rb may not respond to CDK4/6 inhibitors. Moreover, RBNSig-BC’s sensitivity of 100% and specificity of 68.42% in NeoPalAna may also indicate that RBness is necessary but not sufficient for palbociclib resistance, and, in some cases, RBness may be irrelevant to resistance. In summary, as the interplay between the endocrine therapy, CDK4/6 inhibitors and adaptive resistance becomes more precise (*14*, *74*) in adequately powered studies, there exist future opportunities to further refine the use of RBness in patient selection for combination therapies in translational studies.

Despite the paucity of high-throughput mass spectrometry proteomics data for large cancer cohorts, a pan-cancer analysis revealed a common set of RBness-associated genes (RBNSig-core). While the RBNSig-core signature could be used in the preclinical setting to assess RBness for cancer types where proteogenomic RBNSigs are not yet available, the cancer type–specific proteogenomic RBNSigs should be preferred for increased sensitivity and specificity for predicting RBness. The composition of RBNSig-core suggested that a few altered biological pathways could be the universal driving mechanisms behind aggressiveness and sustained proliferation of cancers with RBness. In particular, the increased expression of genes involved in Fanconi anemia core complex and prereplication complex revealed that abnormal DNA replication is likely to codrive RBness together with the canonical cell cycle dysregulation associated to *RB1* dysfunction. While the pan-cancer RBness data can be reconciled on a pathway level, our results also highlight diverse signatures of RBness across cancer types encompassing pathways and genes beyond the cell cycle. For example, interleukin signaling was enriched in genes from RBNSig of LIHC and LUAD only, and the enhancer of zeste homolog 2 (*EZH2*), a histone methyltransferase and catalytic subunit of polycomb repressive complex 2 (PRC2) was specific to RBNSig of LIHC, lung squamous cell carcinoma, and sarcoma. EZH2 catalyzes tri-methylation of histone H3 at Lys^27^ (H3K27me3) and has a role in epigenetic activation and silencing of key oncogenes and tumor suppressor genes in multiple cancers as well as contributing to drug resistance (*75*). Hence, this heterogeneity in cancer type–specific signatures could explain the variable drug responses seen in patient studies and underscores the importance of targeting the right component of the pathway for a given patient to deliver a durable response. For instance, targeting of EZH2, the catalytic subunit of the PRC2, is currently under evaluation in several phase 1/2 clinical trials (*76*) and therefore could potentially offer previously unknown epigenetic therapeutic options for RBNSig-high cancers with EZH2 dysregulation. However, this possibility requires careful consideration since PRC2 is also a tumor suppressor in both breast and lung cancers and leads to context-dependent, phenotypically distinct transitions to mesenchymal state, which rewires the cell cycle and alters their sensitivity to conventional chemotherapy (*77*, *78*).

Previous studies have highlighted *RB1*-dependent sensitivity to AURKA/B inhibitors and also identified AURKB as a synthetic lethal dependency in *RB1*-defective small cell lung cancers (SCLC) (*79*, *80*). However, we did not find *AURKA* or *AURKB* as a synthetic lethal hit in the isogenic *RB1* MCF10A*^TP53−/−^* breast epithelial cell line. This might be influenced by the specific molecular context of the cancer rather than being a universal feature of *RB1* loss. For instance, Gong *et al.* (*79*) observed an up-regulation of spindle-assembly checkpoint (SAC) genes that leads to a primed (activated) state of SAC, as an essential context for the synthetic lethality between *RB1* defects and AURKA inhibition. Similarly, Pearson *et al.* (*81*) observed that retinoblastoma and SCLC that are YAP/TAZ silenced and *RB1* defective are characterized by metabolic dependencies that correlate with high MYC activity as well as by AURK inhibition. These data further highlight additional requirements for the malignant transformation of *RB1*-defective retinoblastoma and SCLCs. Hence, the repurposing potential of AURK inhibitors for RBness needs further work to identify relevant contexts and associated in vitro lineages/models that are suitable for AURK targeting.

In summary, our data show that RBness is not only a transcriptional phenocopy of *RB1*-defective cancers but that it also exhibits clinical characteristics and synthetic lethal vulnerabilities that are known correlates of *RB1* defects. This leads us to predict that additional patients exhibiting RBness in the absence of *RB1* genomic defects could benefit from existing and next-generation treatment strategies designed for *RB1*-defective cancers. For this reason, the identification of an RBness group has translational implications for rationalizing clinical heterogeneity and understanding the unique molecular drivers of treatment resistance and sensitivity. Therefore, this group, alongside bona fide *RB1*-defective patients, also warrants inclusion in preclinical and clinical studies, thereby increasing both statistical power and opportunities to stratify patients into treatment-matched groups. For instance, a logical extension of this work would be to test the efficacy of molecular targeting of RBNSig-associated drug targets such as Fanconi anemia nuclear complex or dual targeting of EHMT2/EZH2 (*82*) in preclinical models that harbor RBness.

## MATERIALS AND METHODS

### TCGA data

Preprocessed mRNA abundance, DNA copy number, somatic mutation profiles and clinical data for TCGA datasets were downloaded from TCGA DCC (gdac), release: 2016_01_28. Genes with >75% zeros across samples for a given cancer type were removed from that cancer type. Copy number calls were based on log_2_ ratio; gain/loss: ±0.3 to ±1, amplification/deep deletion: >±1. For the survival analysis of high-grade OV, clinical stage and grade were grouped into broader categories to improve model fitting since the vast majority of cases in this aggressive cancer type are high stage and grade. For clinical stage, stage ic, stage iia, stage iib, and stage iic were merged into one group, and stage iiia, stage iiib, stage iiic and stage iv were merged into another group. For clinical grade, grades g1 and g2 were merged into one group, and grades g3 and g4 were merged into another group. Although standard-of-care treatment for high-grade OV includes chemotherapy, we identified 34 patients where chemotherapy status was ambiguous and hence these samples were removed from the survival analysis, which was designed to assess chemosensitivity.

### CPTAC data

Preprocessed standardized CPTAC mass spectrometry data (total protein and phosphoprotein) were downloaded from cBioPortal in the form of iTRAQ ratios for BRCA and OV samples and precursor area intensity measurements for the colon and rectal adenocarcinoma (COADREAD) samples (*83*). Preprocessed CPTAC (phase 2/3) mass spectrometry data (total protein and phosphoprotein), mRNA *RB1* genomic data for BRCA samples were downloaded from cBioPortal. Preprocessed CPTAC (phase 2/3) mass spectrometry data (total protein and phosphoprotein) and mRNA data for OV samples were downloaded from the supplementary materials of the original study (*84*), and *RB1* mutation and copy number status was shared by the corresponding authors. Preprocessed CPTAC (phase 2/3) mass spectrometry data (total protein and phosphoprotein) and *RB1* genomic data for LUAD, GBM, and UCEC were downloaded from cBioPortal. Corresponding raw mRNA counts data for LUAD, GBM, and UCEC were downloaded from https://portal.gdc.cancer.gov/.

### SCAN-B data

Normalized RNA-seq data were downloaded from GEO using accession id GSE96058. Sample repeats were removed. Normalized data were exponentiated, and the existing prior of 0.1 was removed and subsequently replaced with a prior of 1, followed by log_2_ transformation. Genes with >75% of samples having zero counts were removed from the dataset.

### Metabric data

The Metabric dataset (*34*) was acquired through formal access made by the Institute of Cancer Research, London, and subsequent processing was performed as detailed previously (*85*).

### BrighTNess trial data

Normalized mRNA abundance and clinical data were downloaded from GEO using accession id GSE164458.

### I-SPY2 trial data

Normalized mRNA abundance data were downloaded from GEO using accession id GSE194040. Clinical data were downloaded from Wolf *et al.* (*43*).

### NeoPalAna trial data

Normalized mRNA abundance data along with probe to gene annotations and clinical annotations was downloaded from GEO using accession id GSE93204.

### Retinoblastoma data

Gene expression data from four retinoblastoma studies were downloaded from GEO using accession ids GSE29683, GSE58780, GSE59983, and GSE196420.

### Discovery of RBNSigs

RBNSigs for BRCA, OV, LUAD, GBM, and UCEC were extracted by combining cancer proteomic, genomic, and transcriptomic data from TCGA (*24*, *48*) and the CPTAC (*25*, *84*, *86*, *87*). A discovery population was identified within the patient cohort of each cancer type/subtype, and included two groups:

(i) *RB1*-defective group: containing those samples displaying Rb abundance ≤ −0.2 (low Rb) or hyperphosphorylation of Rb [pRb ≥0.3 and Rb ≤0.2 and (pRb – Rb) ≥0.2] or having a truncating mutation (MT) in the *RB1* gene or a copy number deep deletion at the *RB1* locus. Missense mutations were excluded from both *RB1*-defective or proficient groups due to uncertainty around the functional consequence of *RB1* missense mutations. pRb was calculated as the median pRb across individual phospho sites for which data were available for a given sample. Using the breast (BRCA) cohort where two CPTAC datasets were available, the threshold for low Rb for the CPTAC phase 2/3 dataset (validation dataset) was set to ≤−0.7 as this ensured matching proportion of low-Rb patients in the discovery and validation datasets. Further, this BRCA-derived threshold was also used to define low Rb for the remainder of the CPTAC phase 2/3 datasets (OV, LUAD, GBM, and UCEC).

(ii) *RB1*-proficient group: containing all samples with no MT(s) in *RB1*, no deep deletion in *RB1*, and having Rb protein abundance >0 (high Rb) as well as mRNA abundance *z* score ≥0.2. This “soft” mRNA abundance filter enables the exclusion of samples potentially harboring an *RB1* loss while showing Rb abundance >0 that would be otherwise considered proficient (Fig. 2A).

The conditions that define *RB1*-defective/*RB1*-proficient samples are strong indicators of the *RB1* dysfunction/functional status in the tumor. Although RPPA data were also available for most of TCGA BRCA samples, mass spectrometry data were preferred because of the stronger correlation between protein and mRNA profiles (fig. S1).

To extract a robust transcriptomic signature from the discovery population, a subsampling procedure was implemented that consisted of rerunning multiple times a differential expression analysis using limma (*88*) while leaving out one sample from the *RB1*-defective group and one sample from the *RB1*-proficient group at a time. Limma was first run on all transcriptomes of the discovery population to identify a set of differentially expressed genes of interest [|log_2_(FC)| ≥0.585 i.e., 1.5 folds in normal scale] and false discovery rate (FDR)–adjusted (Benjamini-Hochberg) *P* value <0.1. Subsequently, the Leave-One-Out-Limma (LOOL) algorithm evaluated the robustness in significance of the selected genes across all n·m unique combinations of (n−1)·(m−1) sample subsets in the discovery population, where n and m are the number of samples from the *RB1*-defective group and the *RB1*-proficient group, respectively. Limma was run using all transcriptomes from the samples in a subset, and the robustness in significance of the selected genes was assessed by counting the fraction of times each of them satisfied certain significance thresholds stricter than the ones previously used [|log_2_(FC)| ≥1 and FDR-adjusted *P* value <0.1]. Exception to this method was for CPTAC-provided raw RNA-seq datasets (LUAD, GBM, and uterine corpus UCEC), where a more suitable Limma-voom was used for differential gene expression analysis and a |log_2_(FC)| ≥0.585 filter was applied during the optimization step.

The final set of differentially expressed genes included all genes that satisfied those thresholds for at least 20% of the combinations and, additionally, showed an average expression (mRNA abundance *z* score) of at least 0.2 in absolute value across the *RB1*-defective samples and a consistent direction in expression in at least 60% of *RB1*-defective samples.

### Signature optimization

To increase the generality of the signatures, a filtering procedure was applied on the basis of a multiscale extraction of genetic relevance networks from the signature genes [Butte AJ *et al.* (*89*)]. Four stepwise increasing values of *RB1* mRNA abundance *z* score were chosen: α∈[0.4, 0.6, 0.8, 1]. For each value of α (*RB1* expression scale), *RB1* characteristic tumors were selected by selecting those samples showing *RB1* expression greater than α or smaller than −α, and a genetic relevance network was then extracted by measuring the correlation coefficient between the mRNA *z* score profiles of the genes across the selected samples (Spearman’s correlation) according to

$$A_{\mathrm{ij}}=\left\{ \begin{array}{c} 1,\;i\neq j\;\text{and}\;C_{\mathrm{ij}}\geq c^{*}\\ 0,\;\text{otherwise} \end{array}\right.$$

where $A_{\mathrm{ij}}$ are the elements of the adjacency matrix describing the network, $C_{\mathrm{ij}}$ is the correlation coefficient between the expression profile of gene i and gene j, and $c^{*}$ is the optimal relevance threshold for which the expression profile of the connected genes in the network (disconnected genes filtered out) were able to best distinguish, in terms of accuracy, the samples with *RB1* expression greater than α from the samples with *RB1* expression smaller than −α. The value of $c^{*}$ was searched heuristically in the range [0.1, 0.8] with steps of 0.02. The classification of samples via the signature was done by computing the alignment score of the connected gene expression to the signature direction for the same genes (see details in subsection Alignment score and signature direction) and grouping them into samples with more than 50% alignment and samples with less than 50% alignment. This procedure was repeated at different scales of *RB1* expression, and a final multiscale consensus of optimized signature genes was identified as the set of genes that were part of the connected network core at all four scales in the case of BRCA and at least two scales in the case of OV (strict optimization for large signatures of more than 50 genes, tolerant optimization for signatures containing less than 50 genes). Figures S2B and S6B show the behavior of the accuracy of sample classification for RBNSig-BC and RBNSig-OV, respectively, expressed as a function of the optimal relevance threshold $c^{*}$ at different expression scales α.

### Alignment score and signature direction

The direction in expression of gene $g_{i}$ in a given sample $s_{j}$ was defined as the mathematical sign of the corresponding *z* score mRNA abundance value $z_{g_{i}}^{s_{j}}$

$$d_{g_{i}}^{s_{j}}=\text{sign}\left(z_{g_{i}}^{s_{j}}\right)$$

The direction of an RBNSig containing N genes labeled $\left\{g_{1},g_{2},\ldots ,g_{N}\right\}$ was defined as the vector containing the signs of the average *z* score mRNA abundance values of each gene measured across the M samples $\left\{s_{1},s_{2},\ldots ,s_{N}\right\}$ of the *RB1*-defective discovery group

$${\vec{d}}_{\text{RBness}}=\text{sign}\left({\bar{z}}_{g_{1}},{\bar{z}}_{g_{2}},\ldots ,{\bar{z}}_{g_{N}}\right)$$

$${\bar{z}}_{g_{i}}=\sum_{\left\{s_{j}\right\}\in \mathrm{RB}1\;\text{defective}}\frac{z_{g_{i}}^{s_{j}}}{M}$$

The alignment score (or simply alignment) of a given sample $s_{j}$ with the signature was defined as the fraction of signature genes in the sample that had a direction concordant to the signature direction for that gene

$$\text{alignment}\left(s_{j},{\vec{d}}_{\text{RBness}}\right)=\sum_{\left\{g_{i}\right\}\in \text{signature}}\frac{d_{g_{i}}^{s_{j}}\cdot d_{\text{RBness}}^{g_{i}}}{N}$$

When the signature was validated on a dataset different from the discovery dataset, only the available genes measured were used to calculate the alignment score.

### Pan-cancer identification of RBNSig

Genomic RBNSigs were extracted from TCGA cancer types where mass spectrometry proteomic data were unavailable. The list of 27 TCGA cancer types is available in table S10. For COADREAD, mass spectrometry proteomics data were available; however, only two samples with associated proteomics had matching *RB1* mRNA abundance RNA-seq data and only two *RB1*-defective samples could be identified with MT of the *RB1* gene, resulting in insufficient sample size for proteogenomic signature discovery.

For each cancer type, a discovery population was identified as the set of patients satisfying the conditions of either of the following two groups:

(i) *RB1-*defective group, containing those samples displaying either an MT (in the absence of proteomics data, we included splice site mutations) at the *RB1* locus, or *RB1* copy number deletion;

(ii) *RB1-*proficient group, containing the top-M samples with highest *z*-score value of *RB1* mRNA abundance that were not members of the *RB1*-defective group, where M is the number of samples in the *RB1*-defective group.

This choice provided a statistical balance in terms of number of samples between the two groups. A list of *RB1*-associated genes was found by running a linear model using limma that identified genes differentially expressed between the two groups with significance thresholds: |log_2_(FC)| ≥0.585 and FDR-adjusted (Benjamini-Hochberg) *P* value <0.1. This was done for all cancer types with at least three samples in the *RB1*-defective group.

Analogous to the methodology used to extract the RBNSigs, the LOOL algorithm was used to select the more robust gene sets with respect to finite population size effects. The thresholds used at each subsampling step to identify significance in differential expression were |log_2_(FC)| ≥1 and FDR-adjusted *P* value <0.1. The final sets of genes included all the genes with average expression larger than 0.2 and at least 60% consistency in direction across the *RB1*-defective samples, which were significantly differentially expressed in at least 50% of the LOOL steps for the cancer types with more than 20 genes initially found, and in at least 20% of the LOOL steps for the cancer types with less than 20 genes initially found. Last, a multiscale gene set optimization was carried out by following the same procedure already described in the Signature optimization subsection by using the tolerant optimization criterion resulting in valid RBNSigs for nine TCGA cancer types (table S10).

### Survival analysis

Survival modeling was performed using Cox proportional hazards model using the R package survival (v3.2-10). Cox models were adjusted for age, tumor size (T-stage), and lymph node status (positive and negative).

### Benchmarking RBNSig-BC

Singular value decomposition (SVD) for each signature was used to generate the signature score. Each gene in the signature was standardized to have mean 0 and SD 1 before performing SVD, and the first eigenvector for the signature was used to estimate SVD. RBNSig-BC and previously published RB-related, E2F targets and proliferation signatures were quantified using the SVD approach for performance comparison. For breast cancer cell lines, *RB1*-defective status was inferred using three datasets: (i) *RB1* MTs were curated from DepMap.org (annotated as “damaging”), (ii) *RB1* deep deletions were curated from cBioPortal.org, and (iii) Rb protein status was curated from Brough *et al.* (*15*).

### RBNSig-core

Genes in the proteogenomic RBNSigs of BRCA, GBM, LUAD, OV, and UCEC, and genomic RBNSigs of BLCA, HNSC, LIHC, LUSC, PRAD, SARC, SKCM, STAD, and UCS were used to extract RBNSig-core. RBNSig-core was defined as the genes that consistently occurred in at least three (20%) of these diseases.

Overlap between cancer type–specific RBNSigs was assessed using the OC given by the formula

$$O\left(A,B\right)=\frac{|A\cap B|}{\text{min}\left(|A|,|B|\right)}$$

*RB1* was excluded from all RBNSigs to adjust for the single count overlap bias.

### Markers of aggressive disease

The hypoxia score for all samples across different cancer types was calculated from the log_2_ RSEM mRNA abundance values of the hypoxia signature genes (*52*) for all TCGA samples of interest using the median of signature genes. The number of clones, somatic mutations, and the tumor purity score were retrieved from TCGA annotation data. The PGA was calculated by estimating the proportion of segmented regions considered as altered (|log_2_ ratio| > 0.2) using TCGA processed segmented data, gdac release 2016_01_28.

### Association with candidate driver alterations

Association between RBNSig alignment scores and candidate somatic driver mutations, amplifications, and deletions was tested using logistic regression. For the pan-cancer model, cancer type was used as a covariate. For the pan-cancer analysis, candidate driver genes were retrieved from COSMIC’s Cancer Gene Census (*58*) that were annotated as “Tier = 1” and “Hallmark = Yes” (download version: 2023-05-16).

### Association with candidate drugs

Preprocessed CTD^2^ data were downloaded from Ben-Hamo *et al.* (*65*). Association between RBNSig-core–predicted groups (low, high-RBness, and *RB1* defective), and z-transformed dose-response area under the receiver operating characteristic curves (dr-AUC) of 481 cancer targeting compounds from the CTD^2^ network (*64*) was tested using Welch’s *t* test. Statistical evaluation was performed for drugs where the mean dr-AUC of RBNSig-core–low group ≥0 and mean dr-AUC of RBNSig-core RBness (or *RB1* defective) group < −0.5.

### CRISPR perturbation screens analysis

Pan-cancer cell lines with available RNA-seq data from CCLE and matched CRISPR gene essentiality profiles were downloaded from the DepMap portal (https://depmap.org/portal/depmap/, version: 21Q4). To lineage-match cell lines with the primary cancer types used for the discovery of RBNSig-core, this analysis was limited to cell lines from these lineages: breast, urinary_tract, central_nervous_system, upper_aerodigestive, liver, lung, prostate, skin, gastric, uterus, ovary, and sarcomas. *RB1*-defective cell lines were defined as those harboring *RB1* deleterious/damaging mutations or *RB1* deep deletion or gene fusions involving *RB1* or *RB1* mRNA abundance *z* score <−2. *RB1* mutation and mRNA data were accessed from depmap.org (version: 21Q4), and copy number and structural variation data (gene fusions) were accessed from cbioportal.org (dated: 2023_02_20). Statistical analyses assessing CRISPR gene effect scores between the *RB1* defective versus RBNSig-low and RBness versus RBNSig-low were performed using two-sample one-sided Welch’s *t* test where a minimum of three observations per group were available. Statistical testing was restricted to genes satisfying the following gene effect (GE) scores criteria

$$\left\{ \begin{array}{c} \text{mean}\left(\text{GE}\;\text{score in group}\;A\right)<-0.5\\ \text{mean}\left(\text{GE}\;\text{score in RBNSig}\;\text{low}\;\text{group}\right)>-0.75\\ \text{mean}\left(\text{GE}\;\text{score in group}\;A\right)<\text{mean}\left(\text{GE}\;\text{score in RBNSig}\;\text{low}\;\text{group}\right) \end{array}\right.$$

where group A is either the *RB1*-defective or RBness group. These thresholds ensured that the mean gene effect in *RB1*-defective or RBness group (< −0.5) represented at least synthetic sickness and remained significantly lower compared to the RBNSig-low group.

Genome-wide CRISPR-Cas9 screen of isogenic MCF10A^*TP53*−/−^ breast epithelial cell line with and without *RB1*^−/−^ was performed using a previously published sgRNA library (Yusa, Sanger) (*90*). Methods pertaining to the generation of *RB1* mutant cells using the MCF10A^*TP53*−/−^ cells and the resulting CRISPR-Cas9 libraries have been described previously here (*91*–*93*). For the prioritization of candidate genes, gene-level sgRNA *z* scores were subjected to the following filters

$$\left\{ \begin{array}{c} \text{Difference}\;\left(\Delta \right):\left(z\;\text{score}\;\mathrm{MCF}10A^{\mathrm{TP}53–/–\mathrm{RB}1–/–}-z\;\text{score}\;\mathrm{MCF}10A^{\mathrm{TP}53–/–}\right)<-2\\ z\;\text{score}\;\mathrm{MCF}10A^{\mathrm{TP}53–/–\mathrm{RB}1–/–}<-2\\ z\;\text{score}\;\mathrm{MCF}10A^{\mathrm{TP}53–/–}>-4 \end{array}\right.$$

### Enrichment analysis

The STRING online analysis tool v.11 (*26*) (https://version-11-0.string-db.org/) was used to perform enrichment analysis of the RBNSig genes for local STRING network clusters, a set of precomputed protein clusters derived by hierarchically clustering the full STRING network via an average linkage algorithm. Default settings were used except for interactions inferred from “Textmining,” which were excluded. The cluster annotations are derived from a consensus of annotations taken from GO, KEGG, REACTOME, UniProt, Pfam, SMART, and InterPro associated to the proteins inside the clusters and can contain combinations of standard annotation terms. The enrichment analysis was performed using a significance threshold of FDR < 0.05. An enrichment network was constructed by defining as nodes all the enriched terms with at least 2 counts and less than 200 overall annotated genes and by connecting nodes that shared at least half of the enriched genes.

Overrepresentation analyses for pathways in REACTOME database as well as elsewhere here were performed using the Fisher’s exact test (R function: phyper). REACTOME pathways were downloaded from MSigDB v7.5.1.

### Data processing, statistical analyses, and visualizations

All data processing, statistical analyses, and plotting were performed in the R statistical environment (v3.6.0).

## References

[1] N. J. Dyson, *RB1*: A prototype tumor suppressor and an enigma. Genes Dev. 30, 1492–1502 (2016). 27401552 10.1101/gad.282145.116 PMC4949322

[2] D. L. Burkhart, J. Sage, Cellular mechanisms of tumour suppression by the retinoblastoma gene. Nat. Rev. Cancer 8, 671–682 (2008). 18650841 10.1038/nrc2399 PMC6996492

[3] F. A. Dick, S. M. Rubin, Molecular mechanisms underlying RB protein function. Nat. Rev. Mol. Cell Biol. 14, 297–306 (2013). 23594950 10.1038/nrm3567 PMC4754300

[4] F. Guzman, Y. Fazeli, M. Khuu, K. Salcido, S. Singh, C. A. Benavente, Retinoblastoma tumor suppressor protein roles in epigenetic regulation. Cancers (Basel). 12, 2807 (2020). 33003565 10.3390/cancers12102807 PMC7600434

[5] M. Chinnam, D. W. Goodrich, *RB1*, development, and cancer. Curr. Top. Dev. Biol. 94, 129–169 (2011). 21295686 10.1016/B978-0-12-380916-2.00005-X PMC3691055

[6] P. Viatour, J. Sage, Newly identified aspects of tumor suppression by RB. Dis. Model. Mech. 4, 581–585 (2011). 21878458 10.1242/dmm.008060 PMC3180221

[7] C. J. Sherr, F. McCormick, The RB and p53 pathways in cancer. Cancer Cell 2, 103–112 (2002). 12204530 10.1016/s1535-6108(02)00102-2

[8] P. Linn, S. Kohno, J. Sheng, N. Kulathunga, H. Yu, Z. Zhang, D. Voon, Y. Watanabe, C. Takahashi, Targeting *RB1* loss in cancers. Cancers (Basel) 13, 3737 (2021). 34359636 10.3390/cancers13153737 PMC8345210

[9] Y. Yao, X. Gu, X. Xu, S. Ge, R. Jia, Novel insights into *RB1* mutation. Cancer Lett. 547, 215870 (2022). 35964818 10.1016/j.canlet.2022.215870

[10] R. Condorelli, L. Spring, J. O'Shaughnessy, L. Lacroix, C. Bailleux, V. Scott, J. Dubois, R. J. Nagy, R. B. Lanman, A. J. Iafrate, F. Andre, A. Bardia, Polyclonal *RB1* mutations and acquired resistance to CDK 4/6 inhibitors in patients with metastatic breast cancer. Ann. Oncol. 29, 640–645 (2018). 29236940 10.1093/annonc/mdx784

[11] M. T. Herrera-Abreu, M. Palafox, U. Asghar, M. A. Rivas, R. J. Cutts, I. Garcia-Murillas, A. Pearson, M. Guzman, O. Rodriguez, J. Grueso, M. Bellet, J. Cortes, R. Elliott, S. Pancholi, J. Baselga, M. Dowsett, L. A. Martin, N. C. Turner, V. Serra, Early adaptation and acquired resistance to CDK4/6 inhibition in estrogen receptor-positive breast cancer. Cancer Res. 76, 2301–2313 (2016). 27020857 10.1158/0008-5472.CAN-15-0728 PMC5426059

[12] K. Pandey, H. J. An, S. K. Kim, S. A. Lee, S. Kim, S. M. Lim, G. M. Kim, J. Sohn, Y. W. Moon, Molecular mechanisms of resistance to CDK4/6 inhibitors in breast cancer: A review. Int. J. Cancer 145, 1179–1188 (2019). 30478914 10.1002/ijc.32020 PMC6767051

[13] E. S. Knudsen, E. Zacksenhaus, The vulnerability of RB loss in breast cancer: Targeting a void in cell cycle control. Oncotarget 9, 30940–30941 (2018). 30123416 10.18632/oncotarget.25797 PMC6089558

[14] A. K. Witkiewicz, S. Chung, R. Brough, P. Vail, J. Franco, C. J. Lord, E. S. Knudsen, Targeting the vulnerability of RB tumor suppressor loss in triple-negative breast cancer. Cell Rep. 22, 1185–1199 (2018). 29386107 10.1016/j.celrep.2018.01.022 PMC5967622

[15] R. Brough, A. Gulati, S. Haider, R. Kumar, J. Campbell, E. Knudsen, S. J. Pettitt, C. J. Ryan, C. J. Lord, Identification of highly penetrant Rb-related synthetic lethal interactions in triple negative breast cancer. Oncogene 37, 5701–5718 (2018). 29915391 10.1038/s41388-018-0368-z PMC6202330

[16] F. A. Dick, D. W. Goodrich, J. Sage, N. J. Dyson, Non-canonical functions of the RB protein in cancer. Nat. Rev. Cancer 18, 442–451 (2018). 29692417 10.1038/s41568-018-0008-5 PMC6693677

[17] W. S. Chen, M. Alshalalfa, S. G. Zhao, Y. Liu, B. A. Mahal, D. A. Quigley, T. Wei, E. Davicioni, T. R. Rebbeck, P. W. Kantoff, C. A. Maher, K. E. Knudsen, E. J. Small, P. L. Nguyen, F. Y. Feng, Novel RB1-Loss transcriptomic signature is associated with poor clinical outcomes across cancer types. Clin. Cancer Res. 25, 4290–4299 (2019). 31010837 10.1158/1078-0432.CCR-19-0404 PMC7883384

[18] A. Ertel, J. L. Dean, H. Rui, C. Liu, A. K. Witkiewicz, K. E. Knudsen, E. S. Knudsen, RB-pathway disruption in breast cancer: Differential association with disease subtypes, disease-specific prognosis and therapeutic response. Cell Cycle 9, 4153–4163 (2010). 20948315 10.4161/cc.9.20.13454 PMC3055199

[19] L. Malorni, S. Piazza, Y. Ciani, C. Guarducci, M. Bonechi, C. Biagioni, C. D. Hart, R. Verardo, A. Di Leo, I. Migliaccio, A gene expression signature of retinoblastoma loss-of-function is a predictive biomarker of resistance to palbociclib in breast cancer cell lines and is prognostic in patients with ER positive early breast cancer. Oncotarget 7, 68012–68022 (2016). 27634906 10.18632/oncotarget.12010 PMC5356535

[20] E. S. Knudsen, R. Nambiar, S. R. Rosario, D. J. Smiraglia, D. W. Goodrich, A. K. Witkiewicz, Pan-cancer molecular analysis of the RB tumor suppressor pathway. Commun. Biol. 3, 158 (2020). 32242058 10.1038/s42003-020-0873-9 PMC7118159

[21] C. J. Lord, A. Ashworth, BRCAness revisited. Nat. Rev. Cancer 16, 110–120 (2016). 26775620 10.1038/nrc.2015.21

[22] C. J. Lord, A. Ashworth, PARP inhibitors: Synthetic lethality in the clinic. Science 355, 1152–1158 (2017). 28302823 10.1126/science.aam7344 PMC6175050

[23] J. Mateo, C. J. Lord, V. Serra, A. Tutt, J. Balmana, M. Castroviejo-Bermejo, C. Cruz, A. Oaknin, S. B. Kaye, J. S. de Bono, A decade of clinical development of PARP inhibitors in perspective. Ann. Oncol. 30, 1437–1447 (2019). 31218365 10.1093/annonc/mdz192 PMC6771225

[24] The Cancer Genome Atlas, Comprehensive molecular portraits of human breast tumours. Nature 490, 61–70 (2012). 23000897 10.1038/nature11412 PMC3465532

[25] K. Krug, E. J. Jaehnig, S. Satpathy, L. Blumenberg, A. Karpova, M. Anurag, G. Miles, P. Mertins, Y. Geffen, L. C. Tang, D. I. Heiman, S. Cao, Y. E. Maruvka, J. T. Lei, C. Huang, R. B. Kothadia, A. Colaprico, C. Birger, J. Wang, Y. Dou, B. Wen, Z. Shi, Y. Liao, M. Wiznerowicz, M. A. Wyczalkowski, X. S. Chen, J. J. Kennedy, A. G. Paulovich, M. Thiagarajan, C. R. Kinsinger, T. Hiltke, E. S. Boja, M. Mesri, A. I. Robles, H. Rodriguez, T. F. Westbrook, L. Ding, G. Getz, K. R. Clauser, D. Fenyö, K. V. Ruggles, B. Zhang, D. R. Mani, S. A. Carr, M. J. Ellis, M. A. Gillette, Clinical Proteomic Tumor Analysis Consortium, Proteogenomic landscape of breast cancer tumorigenesis and targeted therapy. Cell 183, 1436–1456.e31 (2020). 33212010 10.1016/j.cell.2020.10.036 PMC8077737

[26] D. Szklarczyk, A. L. Gable, D. Lyon, A. Junge, S. Wyder, J. Huerta-Cepas, M. Simonovic, N. T. Doncheva, J. H. Morris, P. Bork, L. J. Jensen, C. V. Mering, STRING v11: Protein-protein association networks with increased coverage, supporting functional discovery in genome-wide experimental datasets. Nucleic Acids Res. 47, D607–D613 (2019). 30476243 10.1093/nar/gky1131 PMC6323986

[27] K. Yoshihara, M. Shahmoradgoli, E. Martinez, R. Vegesna, H. Kim, W. Torres-Garcia, V. Trevino, H. Shen, P. W. Laird, D. A. Levine, S. L. Carter, G. Getz, K. Stemke-Hale, G. B. Mills, R. G. Verhaak, Inferring tumour purity and stromal and immune cell admixture from expression data. Nat. Commun. 4, 2612 (2013). 24113773 10.1038/ncomms3612 PMC3826632

[28] J. McEvoy, J. Flores-Otero, J. Zhang, K. Nemeth, R. Brennan, C. Bradley, F. Krafcik, C. Rodriguez-Galindo, M. Wilson, S. Xiong, G. Lozano, J. Sage, L. Fu, L. Louhibi, J. Trimarchi, A. Pani, R. Smeyne, D. Johnson, M. A. Dyer, Coexpression of normally incompatible developmental pathways in retinoblastoma genesis. Cancer Cell 20, 260–275 (2011). 21840489 10.1016/j.ccr.2011.07.005 PMC3551581

[29] J. Liu, D. Ottaviani, M. Sefta, C. Desbrousses, E. Chapeaublanc, R. Aschero, N. Sirab, F. Lubieniecki, G. Lamas, L. Tonon, C. Dehainault, C. Hua, P. Fréneaux, S. Reichman, N. Karboul, A. Biton, L. Mirabal-Ortega, M. Larcher, C. Brulard, S. Arrufat, A. Nicolas, N. Elarouci, T. Popova, F. Némati, D. Decaudin, D. Gentien, S. Baulande, O. Mariani, F. Dufour, S. Guibert, C. Vallot, L. L.-L. Rouic, A. Matet, L. Desjardins, G. Pascual-Pasto, M. Suñol, J. Catala-Mora, G. C. Llano, J. Couturier, E. Barillot, P. Schaiquevich, M. Gauthier-Villars, D. Stoppa-Lyonnet, L. Golmard, C. Houdayer, H. Brisse, I. Bernard-Pierrot, E. Letouzé, A. Viari, S. Saule, X. Sastre-Garau, F. Doz, A. M. Carcaboso, N. Cassoux, C. Pouponnot, O. Goureau, G. Chantada, A. de Reyniès, I. Aerts, F. Radvanyi, A high-risk retinoblastoma subtype with stemness features, dedifferentiated cone states and neuronal/ganglion cell gene expression. Nat. Commun. 12, 5578 (2021). 34552068 10.1038/s41467-021-25792-0 PMC8458383

[30] L. Goldman, J. L. Kenyon, Delays in inactivation development and activation kinetics in myxicola giant axons. J. Gen. Physiol. 80, 83–102 (1982). 6288838 10.1085/jgp.80.1.83 PMC2228670

[31] M. G. Field, J. N. Kuznetsoff, M. G. Zhang, J. J. Dollar, M. A. Durante, Y. Sayegh, C. L. Decatur, S. Kurtenbach, D. Pelaez, J. W. Harbour, RB1 loss triggers dependence on ESRRG in retinoblastoma. Sci. Adv. 8, eabm8466 (2022). 35984874 10.1126/sciadv.abm8466 PMC9390996

[32] H. Liu, Y. Zhang, Y.-Y. Zhang, Y.-P. Li, Z.-Q. Hua, C.-J. Zhang, K.-C. Wu, F. Yu, Y. Zhang, J. Su, Z.-B. Jin, Human embryonic stem cell-derived organoid retinoblastoma reveals a cancerous origin. Proc. Natl. Acad. Sci. U.S.A. 117, 33628–33638 (2020). 33318192 10.1073/pnas.2011780117 PMC7776986

[33] A. K. Witkiewicz, E. S. Knudsen, Retinoblastoma tumor suppressor pathway in breast cancer: Prognosis, precision medicine, and therapeutic interventions. Breast Cancer Res. 16, 207 (2014). 25223380 10.1186/bcr3652 PMC4076637

[34] C. Curtis, S. P. Shah, S.-F. Chin, G. Turashvili, O. M. Rueda, M. J. Dunning, D. Speed, A. G. Lynch, S. Samarajiwa, Y. Yuan, S. Gräf, G. Ha, G. Haffari, A. Bashashati, R. Russell, S. M. Kinney, METABRIC Group, A. Langerød, A. Green, E. Provenzano, G. Wishart, S. Pinder, P. Watson, F. Markowetz, L. Murphy, I. Ellis, A. Purushotham, A.-L. Børresen-Dale, J. D. Brenton, S. Tavaré, C. Caldas, S. Aparicio, The genomic and transcriptomic architecture of 2,000 breast tumours reveals novel subgroups. Nature 486, 346–352 (2012). 22522925 10.1038/nature10983 PMC3440846

[35] N. G. Smith, R. Gyanchandani, O. S. Shah, G. T. Gurda, P. C. Lucas, R. J. Hartmaier, A. M. Brufsky, S. Puhalla, A. Bahreini, K. Kota, A. I. Wald, Y. E. Nikiforov, M. N. Nikiforova, S. Oesterreich, A. V. Lee, Targeted mutation detection in breast cancer using MammaSeq™. Breast Cancer Res. 21, 22 (2019). 30736836 10.1186/s13058-019-1102-7 PMC6368740

[36] V. Theodorou, R. Stark, S. Menon, J. S. Carroll, GATA3 acts upstream of FOXA1 in mediating ESR1 binding by shaping enhancer accessibility. Genome Res. 23, 12–22 (2013). 23172872 10.1101/gr.139469.112 PMC3530671

[37] J. S. Parker, M. Mullins, M. C. Cheang, S. Leung, D. Voduc, T. Vickery, S. Davies, C. Fauron, X. He, Z. Hu, J. F. Quackenbush, I. J. Stijleman, J. Palazzo, J. S. Marron, A. B. Nobel, E. Mardis, T. O. Nielsen, M. J. Ellis, C. M. Perou, P. S. Bernard, Supervised risk predictor of breast cancer based on intrinsic subtypes. J. Clin. Oncol. 27, 1160–1167 (2009). 19204204 10.1200/JCO.2008.18.1370 PMC2667820

[38] D. W. Garsed, K. Alsop, S. Fereday, C. Emmanuel, C. J. Kennedy, D. Etemadmoghadam, B. Gao, V. Gebski, V. Gares, E. L. Christie, M. C. A. Wouters, K. Milne, J. George, A. M. Patch, J. Li, G. M. Arnau, T. Semple, S. R. Gadipally, Y. E. Chiew, J. Hendley, T. Mikeska, G. V. Zapparoli, K. Amarasinghe, S. M. Grimmond, J. V. Pearson, N. Waddell, J. Hung, C. J. R. Stewart, R. Sharma, P. E. Allan, P. F. Rambau, O. McNally, L. Mileshkin, A. Hamilton, S. Ananda, M. Grossi, P. A. Cohen, Y. C. Leung, R. M. Rome, P. Beale, P. Blomfield, M. Friedlander, A. Brand, A. Dobrovic, M. Kobel, P. Harnett, B. H. Nelson, D. D. L. Bowtell, A. deFazio, Nadia Traficante, for the Australian Ovarian Cancer Study Group, Homologous recombination DNA repair pathway disruption and retinoblastoma protein loss are associated with exceptional survival in high-grade serous ovarian cancer. Clin. Cancer Res. 24, 569–580 (2018). 29061645 10.1158/1078-0432.CCR-17-1621

[39] J. I. Herschkowitz, X. He, C. Fan, C. M. Perou, The functional loss of the retinoblastoma tumour suppressor is a common event in basal-like and luminal B breast carcinomas. Breast Cancer Res. 10, R75 (2008). 18782450 10.1186/bcr2142 PMC2614508

[40] E. S. Knudsen, J. Y. Wang, Targeting the RB-pathway in cancer therapy. Clin. Cancer Res. 16, 1094–1099 (2010). 20145169 10.1158/1078-0432.CCR-09-0787 PMC2822892

[41] A. Milea, S. H. George, D. Matevski, H. Jiang, M. Madunic, H. K. Berman, M. L. Gauthier, B. Gallie, P. A. Shaw, Retinoblastoma pathway deregulatory mechanisms determine clinical outcome in high-grade serous ovarian carcinoma. Mod. Pathol. 27, 991–1001 (2014). 24336157 10.1038/modpathol.2013.218

[42] O. M. Filho, D. G. Stover, S. Asad, P. J. Ansell, M. Watson, S. Loibl, C. E. Geyer Jr., J. Bae, K. Collier, M. Cherian, J. O'Shaughnessy, M. Untch, H. S. Rugo, J. B. Huober, M. Golshan, W. M. Sikov, G. von Minckwitz, P. Rastogi, D. Maag, N. Wolmark, C. Denkert, W. F. Symmans, Association of immunophenotype with pathologic complete response to neoadjuvant chemotherapy for triple-negative breast cancer: A secondary analysis of the BrighTNess phase 3 randomized clinical trial. JAMA Oncol. 7, 603–608 (2021). 33599688 10.1001/jamaoncol.2020.7310 PMC7893540

[43] D. M. Wolf, C. Yau, J. Wulfkuhle, L. Brown-Swigart, R. I. Gallagher, P. R. E. Lee, Z. Zhu, M. J. Magbanua, R. Sayaman, N. O’Grady, A. Basu, A. Delson, J. P. Coppé, R. Lu, J. Braun, I-SPY2 Investigators, S. M. Asare, L. Sit, J. B. Matthews, J. Perlmutter, N. Hylton, M. C. Liu, P. Pohlmann, W. F. Symmans, H. S. Rugo, C. Isaacs, A. M. De Michele, D. Yee, D. A. Berry, L. Pusztai, E. F. Petricoin, G. L. Hirst, L. J. Esserman, L. J. van ‘t Veer, Redefining breast cancer subtypes to guide treatment prioritization and maximize response: Predictive biomarkers across 10 cancer therapies. Cancer Cell 40, 609–623.e6 (2022). 35623341 10.1016/j.ccell.2022.05.005 PMC9426306

[44] S. M. Swain, M. Shastry, E. Hamilton, Targeting HER2-positive breast cancer: Advances and future directions. Nat. Rev. Drug Discov. 22, 101–126 (2023). 36344672 10.1038/s41573-022-00579-0 PMC9640784

[45] C. X. Ma, F. Gao, J. Luo, D. W. Northfelt, M. Goetz, A. Forero, J. Hoog, M. Naughton, F. Ademuyiwa, R. Suresh, K. S. Anderson, J. Margenthaler, R. Aft, T. Hobday, T. Moynihan, W. Gillanders, A. Cyr, T. J. Eberlein, T. Hieken, H. Krontiras, Z. Guo, M. V. Lee, N. C. Spies, Z. L. Skidmore, O. L. Griffith, M. Griffith, S. Thomas, C. Bumb, K. Vij, C. H. Bartlett, M. Koehler, H. Al-Kateb, S. Sanati, M. J. Ellis, NeoPalAna: Neoadjuvant palbociclib, a cyclin-dependent kinase 4/6 inhibitor, and anastrozole for clinical stage 2 or 3 estrogen receptor-positive breast cancer. Clin. Cancer Res. 23, 4055–4065 (2017). 28270497 10.1158/1078-0432.CCR-16-3206 PMC5555232

[46] D. Venet, J. E. Dumont, V. Detours, Most random gene expression signatures are significantly associated with breast cancer outcome. PLOS Comput. Biol. 7, e1002240 (2011). 22028643 10.1371/journal.pcbi.1002240 PMC3197658

[47] S. R. McCurdy, M. Pacal, M. Ahmad, R. Bremner, A CDK2 activity signature predicts outcome in CDK2-low cancers. Oncogene 36, 2491–2502 (2017). 27819669 10.1038/onc.2016.409

[48] The Cancer Genome Atlas Research, Integrated genomic analyses of ovarian carcinoma. Nature 474, 609–615 (2011). 21720365 10.1038/nature10166 PMC3163504

[49] B. van Zyl, D. Tang, N. A. Bowden, Biomarkers of platinum resistance in ovarian cancer: What can we use to improve treatment. Endocr. Relat. Cancer 25, R303–R318 (2018). 29487129 10.1530/ERC-17-0336

[50] P. Indovina, F. Pentimalli, N. Casini, I. Vocca, A. Giordano, RB1 dual role in proliferation and apoptosis: Cell fate control and implications for cancer therapy. Oncotarget 6, 17873–17890 (2015). 26160835 10.18632/oncotarget.4286 PMC4627222

[51] C. J. Sherr, Cancer cell cycles. Science 274, 1672–1677 (1996). 8939849 10.1126/science.274.5293.1672

[52] F. M. Buffa, A. L. Harris, C. M. West, C. J. Miller, Large meta-analysis of multiple cancers reveals a common, compact and highly prognostic hypoxia metagene. Br. J. Cancer 102, 428–435 (2010). 20087356 10.1038/sj.bjc.6605450 PMC2816644

[53] S. Haider, S. Tyekucheva, D. Prandi, N. S. Fox, J. Ahn, A. W. Xu, A. Pantazi, P. J. Park, P. W. Laird, C. Sander, W. Wang, F. Demichelis, M. Loda, P. C. Boutros, Cancer Genome Atlas Research Network, Systematic assessment of tumor purity and its clinical implications. JCO Precis. Oncol. 4, PO.20.00016 (2020). 33015524 10.1200/PO.20.00016 PMC7529507

[54] H. Hieronymus, R. Murali, A. Tin, K. Yadav, W. Abida, H. Moller, D. Berney, H. Scher, B. Carver, P. Scardino, N. Schultz, B. Taylor, A. Vickers, J. Cuzick, C. L. Sawyers, Tumor copy number alteration burden is a pan-cancer prognostic factor associated with recurrence and death. eLife 7, e37294 (2018). 30178746 10.7554/eLife.37294 PMC6145837

[55] M. Jamal-Hanjani, S. A. Quezada, J. Larkin, C. Swanton, Translational implications of tumor heterogeneity. Clin. Cancer Res. 21, 1258–1266 (2015). 25770293 10.1158/1078-0432.CCR-14-1429 PMC4374162

[56] L. Ke, S. Li, D. Huang, The predictive value of tumor mutation burden on survival of gastric cancer patients treated with immune checkpoint inhibitors: A systematic review and meta-analysis. Int. Immunopharmacol. 124, 110986 (2023). 37748223 10.1016/j.intimp.2023.110986

[57] L. Li, L. Bai, H. Lin, L. Dong, R. Zhang, X. Cheng, Z. Liu, Y. Ouyang, K. Ding, Multiomics analysis of tumor mutational burden across cancer types. Comput. Struct. Biotechnol. J. 19, 5637–5646 (2021). 34745455 10.1016/j.csbj.2021.10.013 PMC8531462

[58] J. G. Tate, S. Bamford, H. C. Jubb, Z. Sondka, D. M. Beare, N. Bindal, H. Boutselakis, C. G. Cole, C. Creatore, E. Dawson, P. Fish, B. Harsha, C. Hathaway, S. C. Jupe, C. Y. Kok, K. Noble, L. Ponting, C. C. Ramshaw, C. E. Rye, H. E. Speedy, R. Stefancsik, S. L. Thompson, S. Wang, S. Ward, P. J. Campbell, S. A. Forbes, COSMIC: The catalogue of somatic mutations in cancer. Nucleic Acids Res. 47, D941–D947 (2019). 30371878 10.1093/nar/gky1015 PMC6323903

[59] F. M. Behan, F. Iorio, G. Picco, E. Goncalves, C. M. Beaver, G. Migliardi, R. Santos, Y. Rao, F. Sassi, M. Pinnelli, R. Ansari, S. Harper, D. A. Jackson, R. McRae, R. Pooley, P. Wilkinson, D. van der Meer, D. Dow, C. Buser-Doepner, A. Bertotti, L. Trusolino, E. A. Stronach, J. Saez-Rodriguez, K. Yusa, M. J. Garnett, Prioritization of cancer therapeutic targets using CRISPR-Cas9 screens. Nature 568, 511–516 (2019). 30971826 10.1038/s41586-019-1103-9

[60] R. M. Meyers, J. G. Bryan, J. M. McFarland, B. A. Weir, A. E. Sizemore, H. Xu, N. V. Dharia, P. G. Montgomery, G. S. Cowley, S. Pantel, A. Goodale, Y. Lee, L. D. Ali, G. Jiang, R. Lubonja, W. F. Harrington, M. Strickland, T. Wu, D. C. Hawes, V. A. Zhivich, M. R. Wyatt, Z. Kalani, J. J. Chang, M. Okamoto, K. Stegmaier, T. R. Golub, J. S. Boehm, F. Vazquez, D. E. Root, W. C. Hahn, A. Tsherniak, Computational correction of copy number effect improves specificity of CRISPR-Cas9 essentiality screens in cancer cells. Nat. Genet. 49, 1779–1784 (2017). 29083409 10.1038/ng.3984 PMC5709193

[61] Y. Qi, M. Wang, Q. Jiang, PABPC1--mRNA stability, protein translation and tumorigenesis. Front. Oncol. 12, 1025291 (2022). 36531055 10.3389/fonc.2022.1025291 PMC9753129

[62] S. Herkenne, L. Scorrano, OPA1, a new mitochondrial target in cancer therapy. Aging (Albany NY) 12, 20931–20933 (2020). 33216729 10.18632/aging.104207 PMC7695375

[63] S. Pancholi, R. Ribas, N. Simigdala, E. Schuster, J. Nikitorowicz-Buniak, A. Ressa, Q. Gao, M. F. Leal, A. Bhamra, A. Thornhill, L. Morisset, E. Montaudon, L. Sourd, M. Fitzpatrick, M. Altelaar, S. R. Johnston, E. Marangoni, M. Dowsett, L. A. Martin, Tumour kinome re-wiring governs resistance to palbociclib in oestrogen receptor positive breast cancers, highlighting new therapeutic modalities. Oncogene 39, 4781–4797 (2020). 32307447 10.1038/s41388-020-1284-6 PMC7299844

[64] B. A. Aksoy, V. Dancík, K. Smith, J. N. Mazerik, Z. Ji, B. Gross, O. Nikolova, N. Jaber, A. Califano, S. L. Schreiber, D. S. Gerhard, L. C. Hermida, S. Jagu, C. Sander, A. Floratos, P. A. Clemons, CTD2 Dashboard: A searchable web interface to connect validated results from the Cancer Target Discovery and Development Network. Database (Oxford) 2017, bax054 (2017). 29220450 10.1093/database/bax054 PMC5569694

[65] R. Ben-Hamo, A. J. Berger, N. Gavert, M. Miller, G. Pines, R. Oren, E. Pikarsky, C. H. Benes, T. Neuman, Y. Zwang, S. Efroni, G. Getz, R. Straussman, Predicting and affecting response to cancer therapy based on pathway-level biomarkers. Nat. Commun. 11, 3296 (2020). 32620799 10.1038/s41467-020-17090-y PMC7335104

[66] J. Lee, Current treatment landscape for early triple-negative breast cancer (TNBC). J. Clin. Med. 12, 1524 (2023). 36836059 10.3390/jcm12041524 PMC9962369

[67] J. C. Black, E. Atabakhsh, J. Kim, K. M. Biette, C. Van Rechem, B. Ladd, P. D. Burrowes, C. Donado, H. Mattoo, B. P. Kleinstiver, B. Song, G. Andriani, J. K. Joung, O. Iliopoulos, C. Montagna, S. Pillai, G. Getz, J. R. Whetstine, Hypoxia drives transient site-specific copy gain and drug-resistant gene expression. Genes Dev. 29, 1018–1031 (2015). 25995187 10.1101/gad.259796.115 PMC4441050

[68] S. Haider, A. M. Intyre, R. G. P. M. van Stiphout, L. M. Winchester, S. Wigfield, A. L. Harris, F. M. Buffa, Genomic alterations underlie a pan-cancer metabolic shift associated with tumour hypoxia. Genome Biol. 17, 140 (2016). 27358048 10.1186/s13059-016-0999-8 PMC4926297

[69] G. Pons, G. Gallo-Oller, N. Navarro, P. Zarzosa, J. Sansa-Girona, L. Garcia-Gilabert, A. Magdaleno, M. F. Segura, J. de Sanchez Toledo, S. Gallego, L. Moreno, J. Roma, Analysis of cancer genomic amplifications identifies druggable collateral dependencies within the amplicon. Cancers (Basel) 15, 1636 (2023). 36980521 10.3390/cancers15061636 PMC10046350

[70] J. Yang, J. Nie, X. Ma, Y. Wei, Y. Peng, X. Wei, Targeting PI3K in cancer: Mechanisms and advances in clinical trials. Mol. Cancer 18, 26 (2019). 30782187 10.1186/s12943-019-0954-x PMC6379961

[71] M. C. Casimiro, C. Wang, Z. Li, G. Di Sante, N. E. Willmart, S. Addya, L. Chen, Y. Liu, M. P. Lisanti, R. G. Pestell, Cyclin D1 determines estrogen signaling in the mammary gland in vivo. Mol. Endocrinol. 27, 1415–1428 (2013). 23864650 10.1210/me.2013-1065 PMC3753428

[72] R. Lamb, S. Lehn, L. Rogerson, R. B. Clarke, G. Landberg, Cell cycle regulators cyclin D1 and CDK4/6 have estrogen receptor-dependent divergent functions in breast cancer migration and stem cell-like activity. Cell Cycle 12, 2384–2394 (2013). 23839043 10.4161/cc.25403 PMC3841318

[73] D. W. Fry, P. J. Harvey, P. R. Keller, W. L. Elliott, M. Meade, E. Trachet, M. Albassam, X. Zheng, W. R. Leopold, N. K. Pryer, P. L. Toogood, Specific inhibition of cyclin-dependent kinase 4/6 by PD 0332991 and associated antitumor activity in human tumor xenografts. Mol. Cancer Ther. 3, 1427–1438 (2004). 15542782

[74] P. J. Roberts, V. Kumarasamy, A. K. Witkiewicz, E. S. Knudsen, Chemotherapy and CDK4/6 inhibitors: Unexpected bedfellows. Mol. Cancer Ther. 19, 1575–1588 (2020). 32546660 10.1158/1535-7163.MCT-18-1161 PMC7473501

[75] L. Gan, Y. Yang, Q. Li, Y. Feng, T. Liu, W. Guo, Epigenetic regulation of cancer progression by EZH2: From biological insights to therapeutic potential. Biomark Res. 6, 10 (2018). 29556394 10.1186/s40364-018-0122-2 PMC5845366

[76] R. Duan, W. Du, W. Guo, EZH2: A novel target for cancer treatment. J. Hematol. Oncol. 13, 104 (2020). 32723346 10.1186/s13045-020-00937-8 PMC7385862

[77] M. Serresi, B. Siteur, D. Hulsman, C. Company, M. J. Schmitt, C. Lieftink, B. Morris, M. Cesaroni, N. Proost, R. L. Beijersbergen, M. van Lohuizen, G. Gargiulo, Ezh2 inhibition in Kras-driven lung cancer amplifies inflammation and associated vulnerabilities. J. Exp. Med. 215, 3115–3135 (2018). 30487290 10.1084/jem.20180801 PMC6279402

[78] Y. Zhang, J. L. Donaher, S. Das, X. Li, F. Reinhardt, J. A. Krall, A. W. Lambert, P. Thiru, H. R. Keys, M. Khan, M. Hofree, M. M. Wilson, O. Yedier-Bayram, N. A. Lack, T. T. Onder, T. Bagci-Onder, M. Tyler, I. Tirosh, A. Regev, J. A. Lees, R. A. Weinberg, Genome-wide CRISPR screen identifies PRC2 and KMT2D-COMPASS as regulators of distinct EMT trajectories that contribute differentially to metastasis. Nat. Cell Biol. 24, 554–564 (2022). 35411083 10.1038/s41556-022-00877-0 PMC9037576

[79] X. Gong, J. Du, S. H. Parsons, F. F. Merzoug, Y. Webster, P. W. Iversen, L.-C. Chio, R. D. Van Horn, X. Lin, W. Blosser, B. Han, S. Jin, S. Yao, H. Bian, C. Ficklin, L. Fan, A. Kapoor, S. Antonysamy, A. M. M. Nulty, K. Froning, D. Manglicmot, A. Pustilnik, K. Weichert, S. R. Wasserman, M. Dowless, C. Marugán, C. Baquero, M. J. Lallena, S. W. Eastman, Y.-H. Hui, M. Z. Dieter, T. Doman, S. Chu, H.-R. Qian, X. S. Ye, D. A. Barda, G. D. Plowman, C. Reinhard, R. M. Campbell, J. R. Henry, S. G. Buchanan, Aurora A kinase inhibition is synthetic lethal with loss of the RB1 tumor suppressor gene. Cancer Discov. 9, 248–263 (2019). 30373917 10.1158/2159-8290.CD-18-0469

[80] M. G. Oser, R. Fonseca, A. A. Chakraborty, R. Brough, A. Spektor, R. B. Jennings, A. Flaifel, J. S. Novak, A. Gulati, E. Buss, S. T. Younger, S. K. McBrayer, G. S. Cowley, D. M. Bonal, Q. D. Nguyen, L. Brulle-Soumare, P. Taylor, S. Cairo, C. J. Ryan, E. J. Pease, K. Maratea, J. Travers, D. E. Root, S. Signoretti, D. Pellman, S. Ashton, C. J. Lord, S. T. Barry, W. G. Kaelin Jr., Cells lacking the RB1 tumor suppressor gene are hyperdependent on Aurora B kinase for survival. Cancer Discov. 9, 230–247 (2019). 30373918 10.1158/2159-8290.CD-18-0389 PMC6368871

[81] J. D. Pearson, K. Huang, M. Pacal, S. R. M. Curdy, S. Lu, A. Aubry, T. Yu, K. M. Wadosky, L. Zhang, T. Wang, A. Gregorieff, M. Ahmad, H. Dimaras, E. Langille, S. P. C. Cole, P. P. Monnier, B. H. Lok, M.-S. Tsao, N. Akeno, D. Schramek, K. A. Wikenheiser-Brokamp, E. S. Knudsen, A. K. Witkiewicz, J. L. Wrana, D. W. Goodrich, R. Bremner, Binary pan-cancer classes with distinct vulnerabilities defined by pro- or anti-cancer YAP/TEAD activity. Cancer Cell 39, 1115–1134.e12 (2021). 34270926 10.1016/j.ccell.2021.06.016 PMC8981970

[82] X. Yang, L. Xu, L. Yang, Recent advances in EZH2-based dual inhibitors in the treatment of cancers. Eur. J. Med. Chem. 256, 115461 (2023). 37156182 10.1016/j.ejmech.2023.115461

[83] P. Wu, Z. J. Heins, J. T. Muller, L. Katsnelson, I. de Bruijn, A. A. Abeshouse, N. Schultz, D. Fenyo, J. Gao, Integration and analysis of CPTAC proteomics data in the context of cancer genomics in the cBioPortal. Mol. Cell. Proteomics 18, 1893–1898 (2019). 31308250 10.1074/mcp.TIR119.001673 PMC6731080

[84] J. E. M. Dermott, O. A. Arshad, V. A. Petyuk, Y. Fu, M. A. Gritsenko, T. R. Clauss, R. J. Moore, A. A. Schepmoes, R. Zhao, M. E. Monroe, M. Schnaubelt, C.-F. Tsai, S. H. Payne, C. Huang, L.-B. Wang, S. Foltz, M. Wyczalkowski, Y. Wu, E. Song, M. A. Brewer, M. Thiagarajan, C. R. Kinsinger, A. I. Robles, E. S. Boja, H. Rodriguez, D. W. Chan, B. Zhang, Z. Zhang, L. Ding, R. D. Smith, T. Liu, K. D. Rodland, Clinical Proteomic Tumor Analysis Consortium, Proteogenomic characterization of ovarian HGSC implicates mitotic kinases, replication stress in observed chromosomal instability. Cell Rep. Med. 1, 100004 (2020). 32529193 10.1016/j.xcrm.2020.100004 PMC7289043

[85] S. Haider, C. Q. Yao, V. S. Sabine, M. Grzadkowski, V. Stimper, M. H. W. Starmans, J. Wang, F. Nguyen, N. C. Moon, X. Lin, C. Drake, C. A. Crozier, C. L. Brookes, C. J. H. van de Velde, A. Hasenburg, D. G. Kieback, C. J. Markopoulos, L. Y. Dirix, C. Seynaeve, D. W. Rea, A. Kasprzyk, P. Lambin, P. Lio, J. M. S. Bartlett, P. C. Boutros, Pathway-based subnetworks enable cross-disease biomarker discovery. Nat. Commun. 9, 4746 (2018). 30420699 10.1038/s41467-018-07021-3 PMC6232113

[86] P. Mertins, D. R. Mani, K. V. Ruggles, M. A. Gillette, K. R. Clauser, P. Wang, X. Wang, J. W. Qiao, S. Cao, F. Petralia, E. Kawaler, F. Mundt, K. Krug, Z. Tu, J. T. Lei, M. L. Gatza, M. Wilkerson, C. M. Perou, V. Yellapantula, K. L. Huang, C. Lin, M. D. McLellan, P. Yan, S. R. Davies, R. R. Townsend, S. J. Skates, J. Wang, B. Zhang, C. R. Kinsinger, M. Mesri, H. Rodriguez, L. Ding, A. G. Paulovich, D. Fenyo, M. J. Ellis, S. A. Carr, C. Nci, Proteogenomics connects somatic mutations to signalling in breast cancer. Nature 534, 55–62 (2016). 27251275 10.1038/nature18003 PMC5102256

[87] H. Zhang, T. Liu, Z. Zhang, S. H. Payne, B. Zhang, J. E. M. Dermott, J.-Y. Zhou, V. A. Petyuk, L. Chen, D. Ray, S. Sun, F. Yang, L. Chen, J. Wang, P. Shah, S. W. Cha, P. Aiyetan, S. Woo, Y. Tian, M. A. Gritsenko, T. R. Clauss, C. Choi, M. E. Monroe, S. Thomas, S. Nie, C. Wu, R. J. Moore, K.-H. Yu, D. L. Tabb, D. Fenyö, V. Bafna, Y. Wang, H. Rodriguez, E. S. Boja, T. Hiltke, R. C. Rivers, L. Sokoll, H. Zhu, I.-M. Shih, L. Cope, A. Pandey, B. Zhang, M. P. Snyder, D. A. Levine, R. D. Smith, D. W. Chan, K. D. Rodland, CPTAC Investigators, Integrated proteogenomic characterization of human high-grade serous ovarian cancer. Cell 166, 755–765 (2016). 27372738 10.1016/j.cell.2016.05.069 PMC4967013

[88] M. E. Ritchie, B. Phipson, D. Wu, Y. Hu, C. W. Law, W. Shi, G. K. Smyth, limma powers differential expression analyses for RNA-sequencing and microarray studies. Nucleic Acids Res. 43, e47 (2015). 25605792 10.1093/nar/gkv007 PMC4402510

[89] A Butte, I. Kohane, *Relevance networks: A first step toward finding genetic regulatory networks within microarray data* in *The Analysis of Gene Expression Data: Methods and Software*, G. Parmigiani, E. S. Garrett, R. A. Irizarry, S. L. Zeger, Eds. (Springer, 2003) 428–446.

[90] H. Koike-Yusa, Y. Li, E.-P. Tan, M. D. C. Velasco-Herrera, K. Yusa, Genome-wide recessive genetic screening in mammalian cells with a lentiviral CRISPR-guide RNA library. Nat. Biotechnol. 32, 267–273 (2014). 24535568 10.1038/nbt.2800

[91] S. Kim, D. Kim, S. W. Cho, J. Kim, J. S. Kim, Highly efficient RNA-guided genome editing in human cells via delivery of purified Cas9 ribonucleoproteins. Genome Res. 24, 1012–1019 (2014). 24696461 10.1101/gr.171322.113 PMC4032847

[92] M. J. Llorca-Cardenosa, L. I. Aronson, D. B. Krastev, J. Nieminuszczy, J. Alexander, F. Song, M. Dylewska, R. Broderick, R. Brough, A. Zimmermann, F. T. Zenke, B. Gurel, R. Riisnaes, A. Ferreira, T. Roumeliotis, J. Choudhary, S. J. Pettitt, J. de Bono, A. Cervantes, S. Haider, W. Niedzwiedz, C. J. Lord, I. Y. Chong, SMG8/SMG9 heterodimer loss modulates SMG1 kinase to drive ATR inhibitor resistance. Cancer Res. 82, 3962–3973 (2022). 36273494 10.1158/0008-5472.CAN-21-4339 PMC9627126

[93] S. Haider, R. Brough, S. Madera, J. Iacovacci, A. Gulati, A. Wicks, J. Alexander, S. J. Pettitt, A. N. J. Tutt, C. J. Lord, The transcriptomic architecture of common cancers reflects synthetic lethal interactions. Nat. Genet., 10.1038/s41588-025-02108-2 (2025). PMC11906352 40033056

