# Supplementary material for: Proteogenomic discovery of RB1-defective phenocopy in cancer predicts disease outcome, response to treatment, and therapeutic targets
Source: Sci Adv. 2025 Mar 26;11(13):eadq9495. doi: 10.1126/sciadv.adq9495 (PMC11939072; doi:10.1126/sciadv.adq9495)
Supplement: Supplementary file 1 — Figs. S1 to S13 Legends for tables S1 to S18 [file sciadv.adq9495_sm.pdf]

Science Advances
AAAS

Supplementary Materials for

Proteogenomic discovery of *RB1*-defective phenocopy in cancer predicts disease outcome, response to treatment, and therapeutic targets

Jacopo Iacovacci *et al.*

Corresponding author: Christopher J. Lord, chris.lord@icr.ac.uk; Syed Haider, syed.haider@icr.ac.uk

Sci. Adv. **11**, eadq9495 (2025)
DOI: 10.1126/sciadv.adq9495

The PDF file includes:

Figs. S1 to S13
Legends for tables S1 to S18

Other Supplementary Material for this manuscript includes the following:

Tables S1 to S18

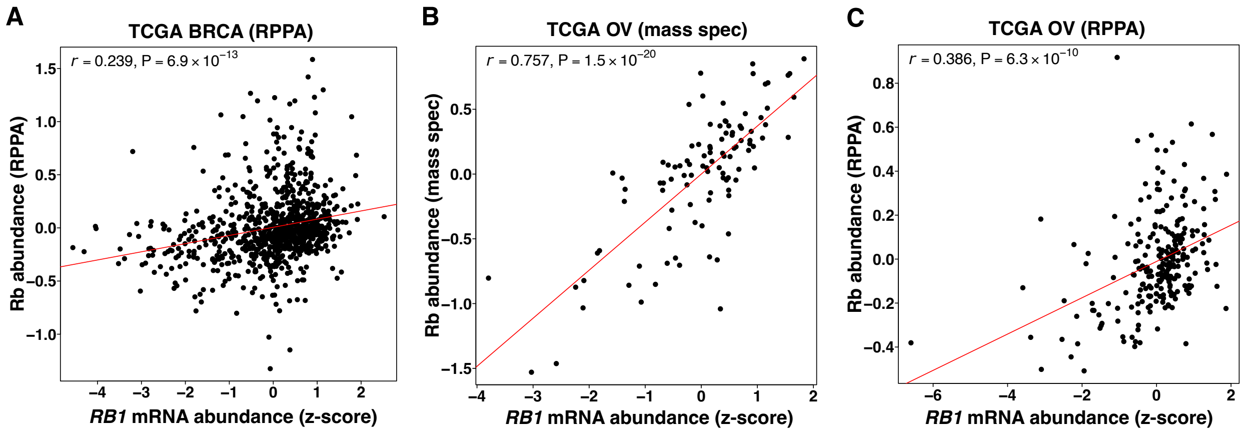

Fig S1. Platform comparison of Rb protein abundance profiles in TCGA breast (BRCA) and ovarian (OV) cancers

(A) Scatter plot showing Pearson's correlation between *RB1* mRNA abundance and Rb protein abundance measured using the reverse-phase protein microarray (RPPA) platform in TCGA BRCA dataset. **(B, C)** Scatter plot showing correlation between *RB1* mRNA abundance and Rb protein abundance measured using the mass-spectrometry (B) and the RPPA (C) platforms in TCGA OV dataset.

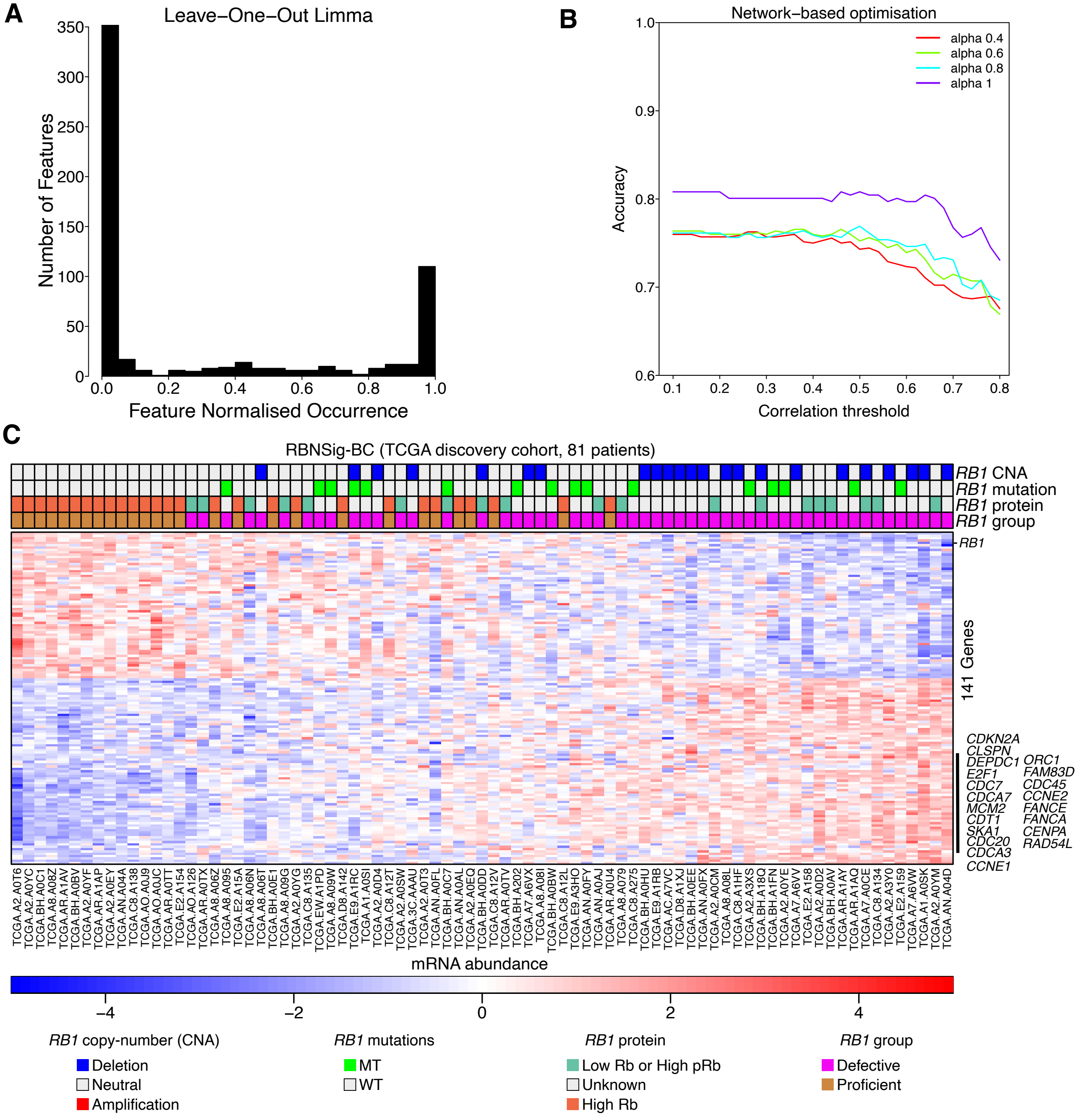

Fig S2. Discovery of RBNSig-BC

(A) Occurrence of significant differentially expressed features in the signature after Limma was applied using an iterative leave-one-out procedure to the TCGA discovery population for RBNSig-BC. **(B)** Accuracy of the core of correlated signature features in classifying the entire TCGA population on the basis of *RB1* mRNA abundance at different expression scales (z-score < -alpha, z-score > alpha) as a function of the correlation threshold, for RBNSig-BC. **(C)** Heatmap showing

mRNA abundance of RBNSig-BC genes in TCGA discovery cohort. mRNA abundance is shown as z-scores across patients. *RB1* and key genes up-regulated in *RB1*-defective cancers are highlighted. MT denotes *RB1* truncating mutations and WT denote wild type *RB1*.

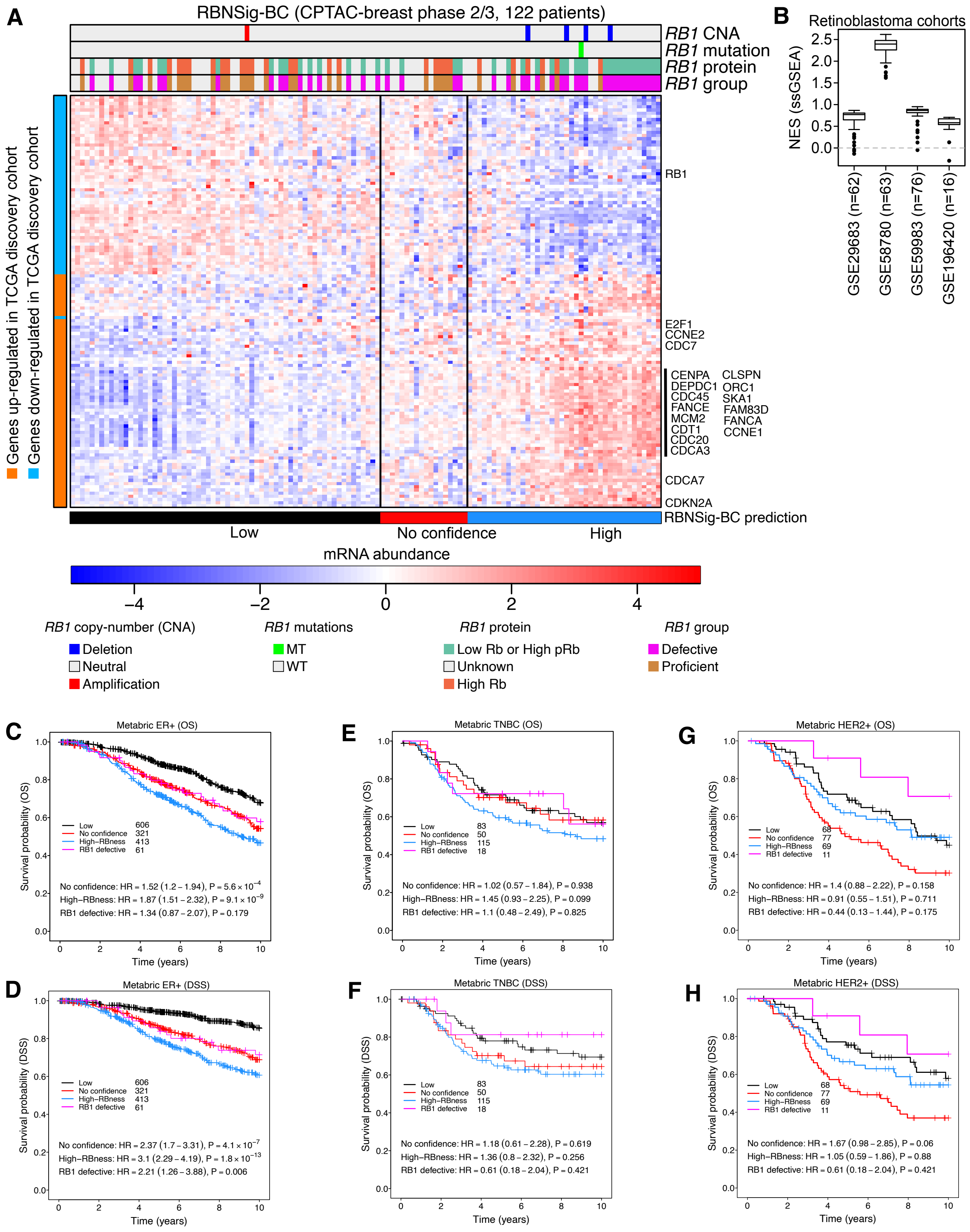

Fig S3. Independent validation of RBNSig-BC

(A) Heatmap showing mRNA abundance of RBNSig-BC genes in CPTAC-breast phase 2/3 cohort. mRNA abundance is shown as z-scores across patients. *RB1* and key genes up-regulated in *RB1*-defective cancers are highlighted. MT denotes *RB1* truncating mutations and WT denote wild type *RB1*. **(B)** Single sample normalised enrichment scores (NES) of genes up-regulated in RBNSig-BC across four retinoblastoma gene expression datasets. Number of samples in each dataset are shown in parenthesis. Values above zero indicate over expression of signature genes in retinoblastoma. **(C, D)** Prognostic assessment of RBNSig-BC in Metabric ER+ cohort using overall survival (OS, C) and disease specific survival (DSS, D). **(E, F)** Prognostic assessment of RBNSig-BC in Metabric TNBC cohort using overall survival (OS, E) and disease specific survival (DSS, F). **(G, H)** Prognostic assessment of RBNSig-BC in Metabric HER2+ cohort using overall survival (OS, G) and disease specific survival (DSS, H). For C-H, multivariable Cox proportional hazards model was adjusted for age, tumour size (T-stage) and lymph node status.

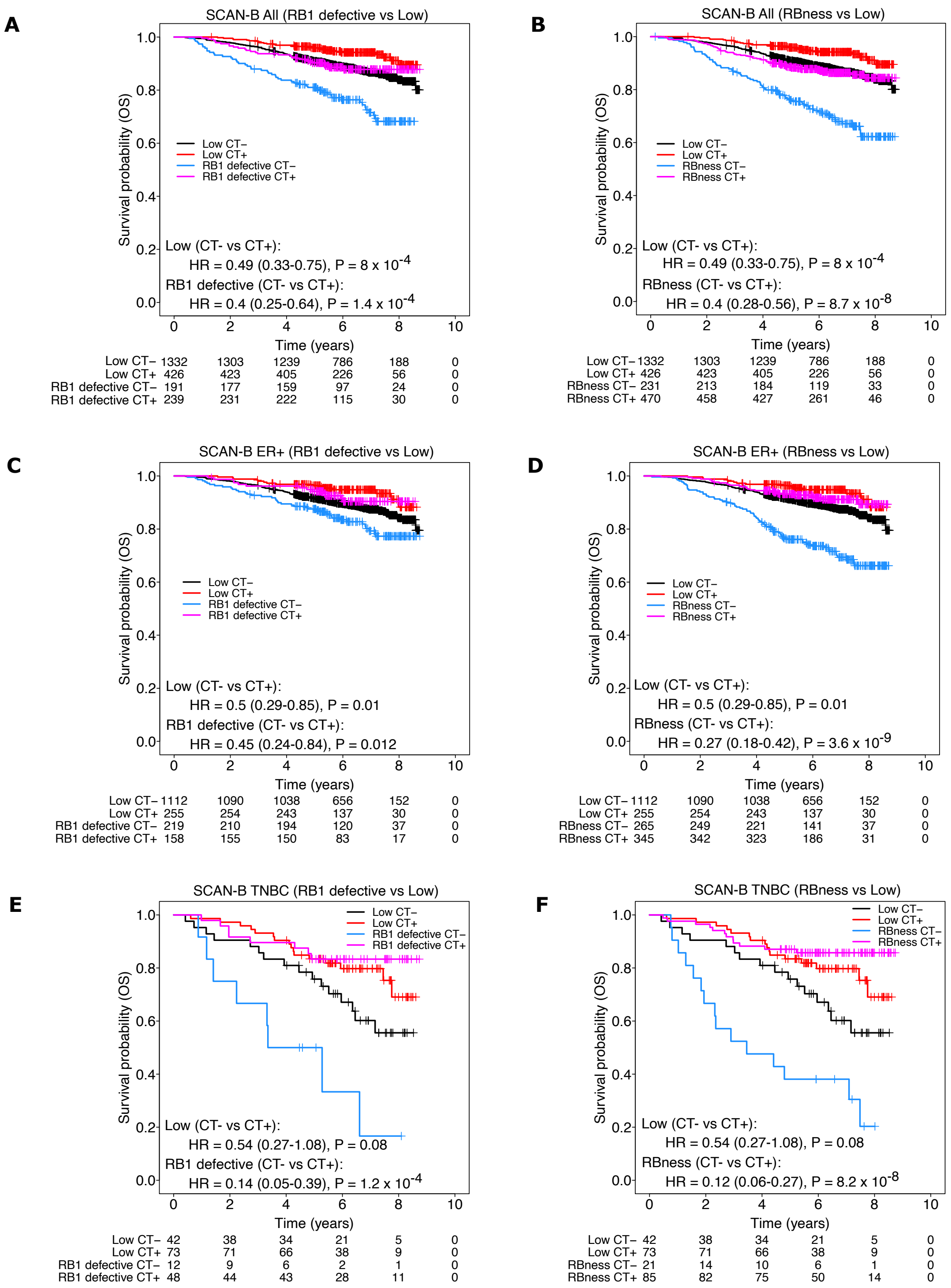

Fig S4. Prognostic assessment of RBNSig-BC predictions in SCAN-B cohort stratified by chemotherapy

(A-F) Prognostic assessment of RBNSig-BC predicted groups in all breast cancer patients, and ER+ and TNBC subtypes, from the SCAN-B cohort. Predicted groups were further stratified by

chemotherapy status: CT- = chemo-naïve, CT+ = chemo-treated. **(A-B)** shows data on all breast cancer patients. **(C-D)** shows data from ER+ patients only. **(E-F)** shows data from TNBC patients only.

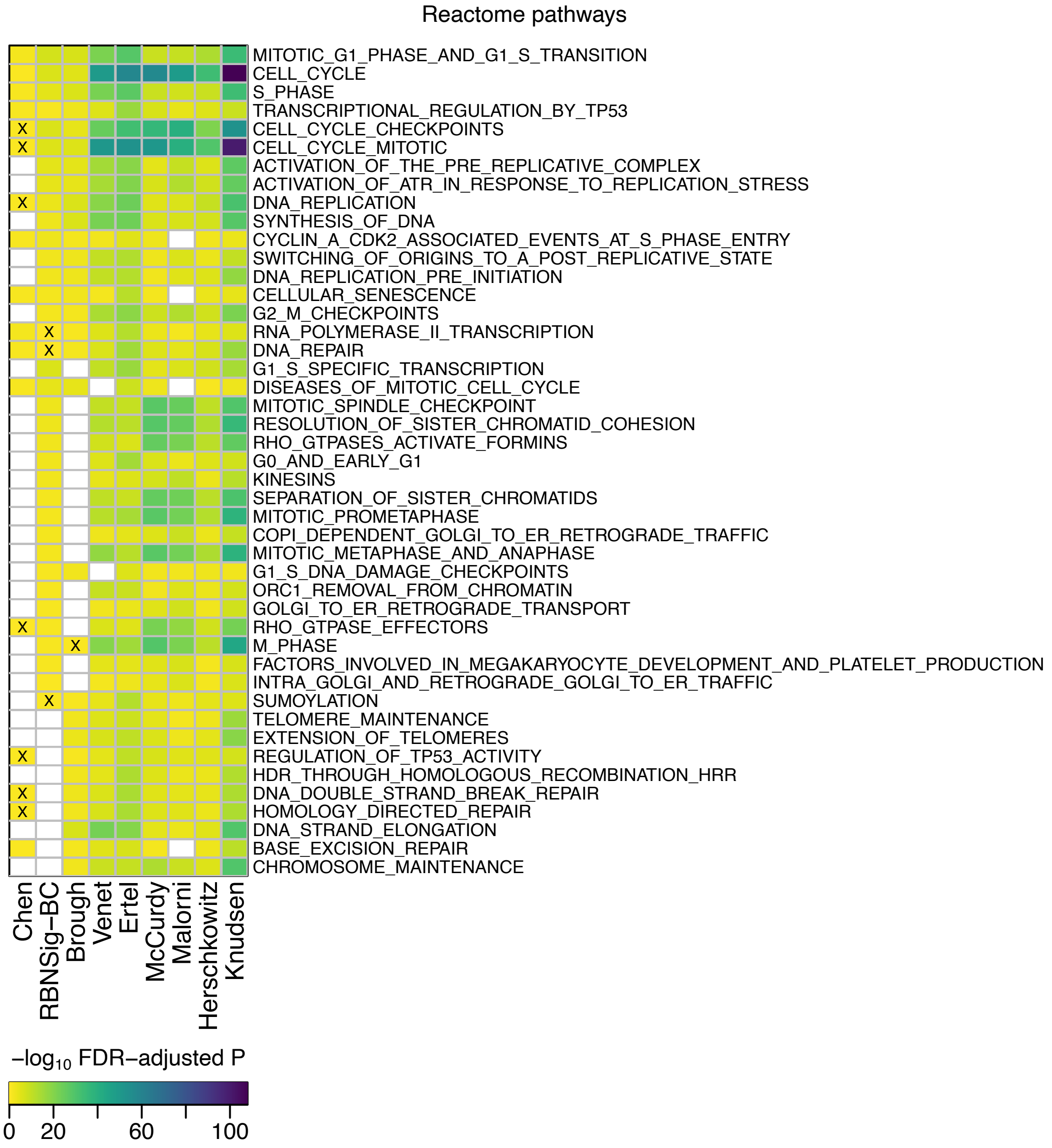

Fig S5. Pathway enrichment analysis of RB-related gene signatures

Heatmap showing significance of recurrently enriched REACTOME pathways. A pathway was considered recurrent if it was significantly enriched (over-representation FDR-adjusted P<0.1; Fisher's exact test) in at least 75% (7 out of 9) of the signatures. White boxes represent NA values

where over-representation analysis was not performed due to overlap of less than three genes. ‘X’ indicates insignificant FDR-adjusted P (< 0.1).

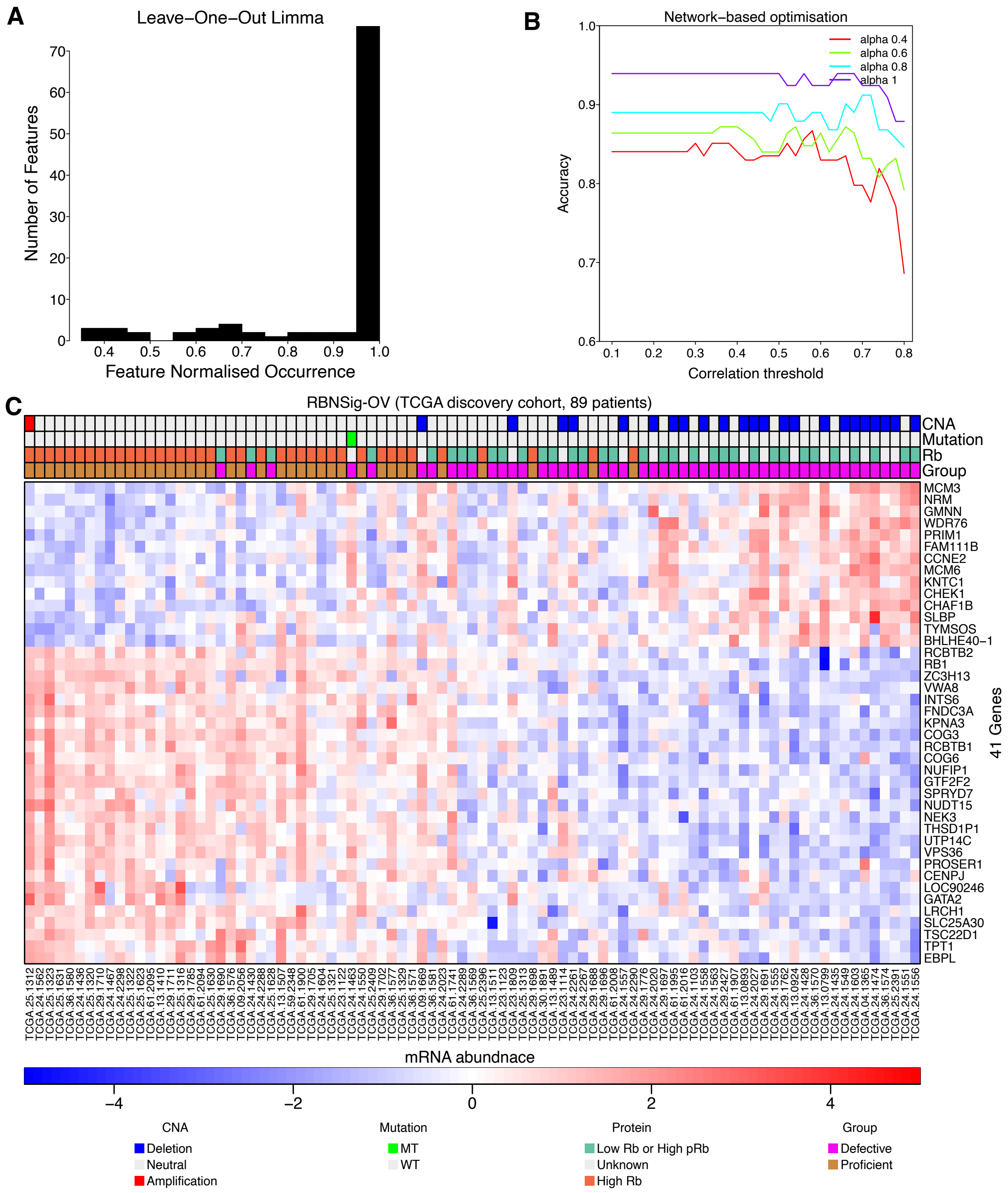

Fig S6. Discovery of RBNSig-OV

(A) Occurrence of significant differentially expressed features in the signature after Limma was applied using an iterative leave-one-out procedure to the TCGA discovery population for RBNSig-

OV. **(B)** Accuracy of the core of correlated signature features in classifying the entire TCGA population on the basis of *RB1* mRNA abundance at different expression scales (z-score < -alpha, z-score > alpha) as a function of the correlation threshold, for RBNSig-OV. **(C)** Heatmap showing mRNA abundance of RBNSig-OV genes in TCGA discovery cohort. mRNA abundance is shown as z-scores across patients. MT denotes *RB1* truncating mutations and WT denote wild type *RB1*.

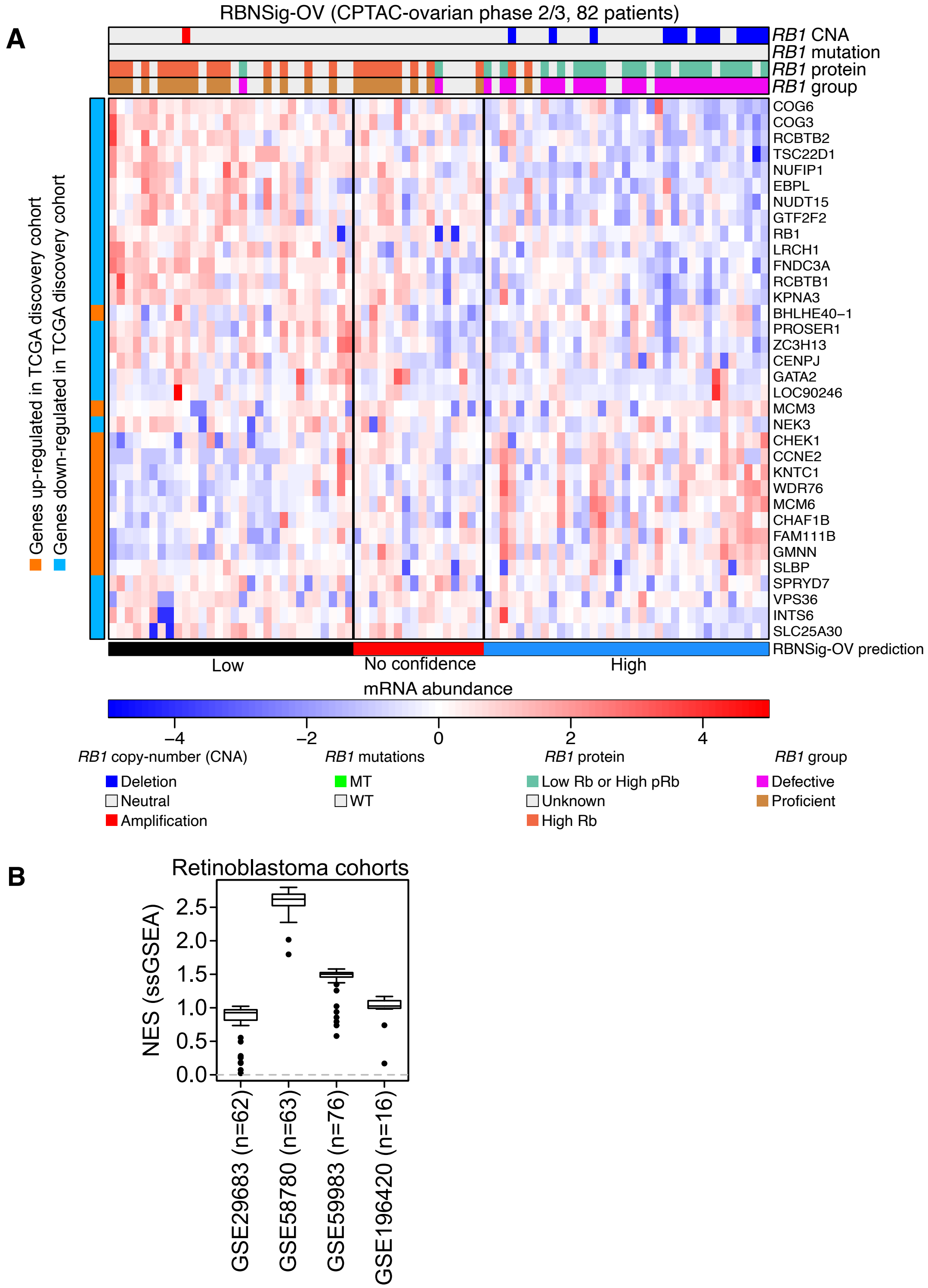

Fig S7. Independent validation of RBNSig-OV

(A) Heatmap showing mRNA abundance of RBNSig-OV genes in CPTAC-ovarian phase 2/3 cohort. mRNA abundance is shown as z-scores across patients. MT denotes *RB1* truncating mutations and WT denote wild type *RB1*. **(B)** Single sample normalised enrichment scores (NES)

of genes up-regulated in RBNSig-OV across four retinoblastoma gene expression datasets. Number of samples in each dataset are shown in parenthesis. Values above zero indicate over expression of signature genes in retinoblastoma.

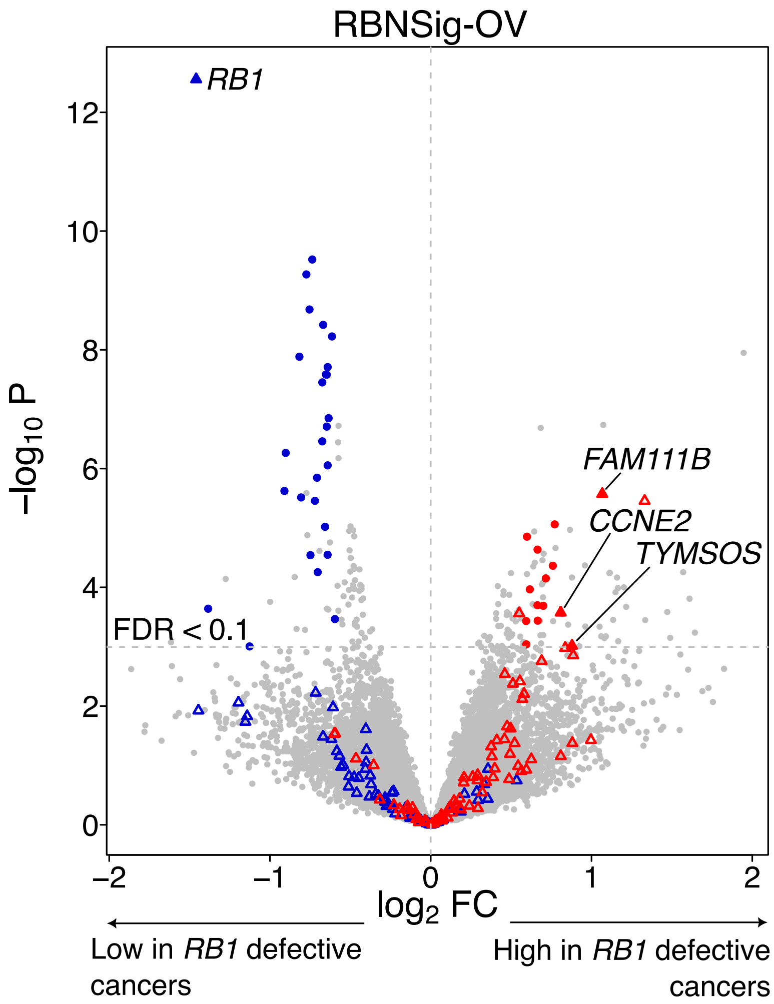

Fig S8. RBNSig-BC genes projected on differential expression statistics of RBNSig-OV

Volcano plot showing differential gene expression results between *RB1*-defective and *RB1*-proficient cancers in TCGA ovarian cohort. Genes highlighted in red and blue circles represent genes included in RBNSig-OV. Genes highlighted in red and blue triangles represent genes included in the breast cancer signature (RBNSig-BC). Filled triangles indicate three genes that were shared between the two signatures.

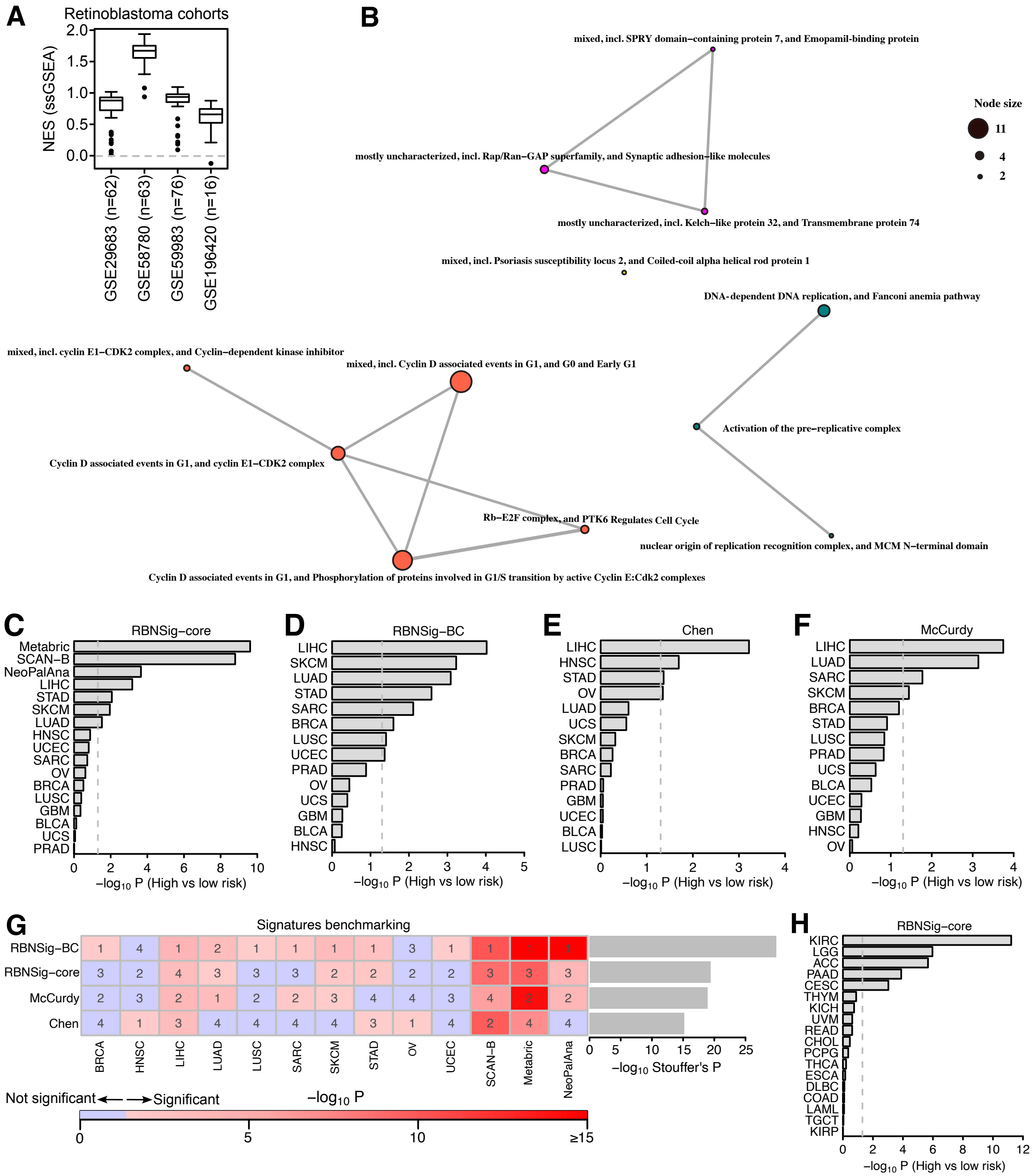

Fig S9. Characterisation and benchmarking of RBNSig-core

(A) Single sample normalised enrichment scores (NES) of genes up-regulated in RBNSig-core across four retinoblastoma gene expression datasets. Number of samples in each dataset are shown in parenthesis. Values above zero indicate over expression of signature genes in retinoblastoma. **(B)** STRING network clusters significantly enriched for genes in RBNSig-core (FDR-adjusted P < 0.05). Size of the node is proportional to the number of enriched genes, and a link between two

clusters exists if there is at least 50% overlap between the sets of enriched genes they included. **(C)** Prognostic (overall survival) and predictive (response to CDK4/6i) performance assessment of RBNSig-core in TCGA cancer types and breast cancer cohorts (Metabric, SCAN-B and NeoPalAna). RBNSig-core predicted per-patient risk score was estimated using Singular value decomposition (Methods: Benchmarking signatures). Risk scores were trichotomized using tertiles, and low and high groups were compared using the Cox proportional hazards model. Wald-test P value was used to assess a signature's prognostic ability. For NeoPalAna, response to CDK4/6i was assessed using AUC-ROC. **(D-F)** Prognostic performance assessment of RBNSig-BC, a pan-cancer *RB1* loss signature (Chen et al. *(17)*) and a pan-cancer *CDK2* activity signature (McCurdy et al. *(47)*) in the 14 TCGA cancer types that contributed to the development of RBNSig-core. **(G)** Performance comparison of RBNSig-BC, RBNSig-core, a previously published pan-cancer *RB1* loss signature (Chen et al. *(17)*) and a previously published *CDK2* activity signature in 14 TCGA cancer datasets, Metabric and SCAN-B breast cancer cohorts, and NeoPalAna breast cancer trial; 17 datasets in total. Four cancer types (BLCA, GBM, PRAD and UCS) where all four signatures were not significantly associated with patient outcome were excluded from this comparison. Heatmap showing significance of association between RBNSig-predicted low and high groups and patient outcome data (overall survival for all datasets except for the NeoPalAna where treatment response to CDK4/6 inhibitor was used). Each signature's risk scores were trichotomized using tertiles, and low and high groups were compared using the Cox proportional hazards model. Wald-test P value was used to assess the signature's performance. Numbers in cells show the rank (across a column) of each signature compared to other signatures for each dataset. Bar plot shows combined weighted (accounting for dataset size) P-value using Stouffer's method. **(H)** Prognostic performance assessment of RBNSig-core in 18 TCGA cancer types that lacked frequent genomic *RB1* defects. RBNSig-core predicted per-patient risk score was estimated using Singular value decomposition. Risk scores were trichotomized using tertiles, and low and high groups were compared using the Cox proportional hazards model. Wald-test P value was used to assess a signature's prognostic ability.

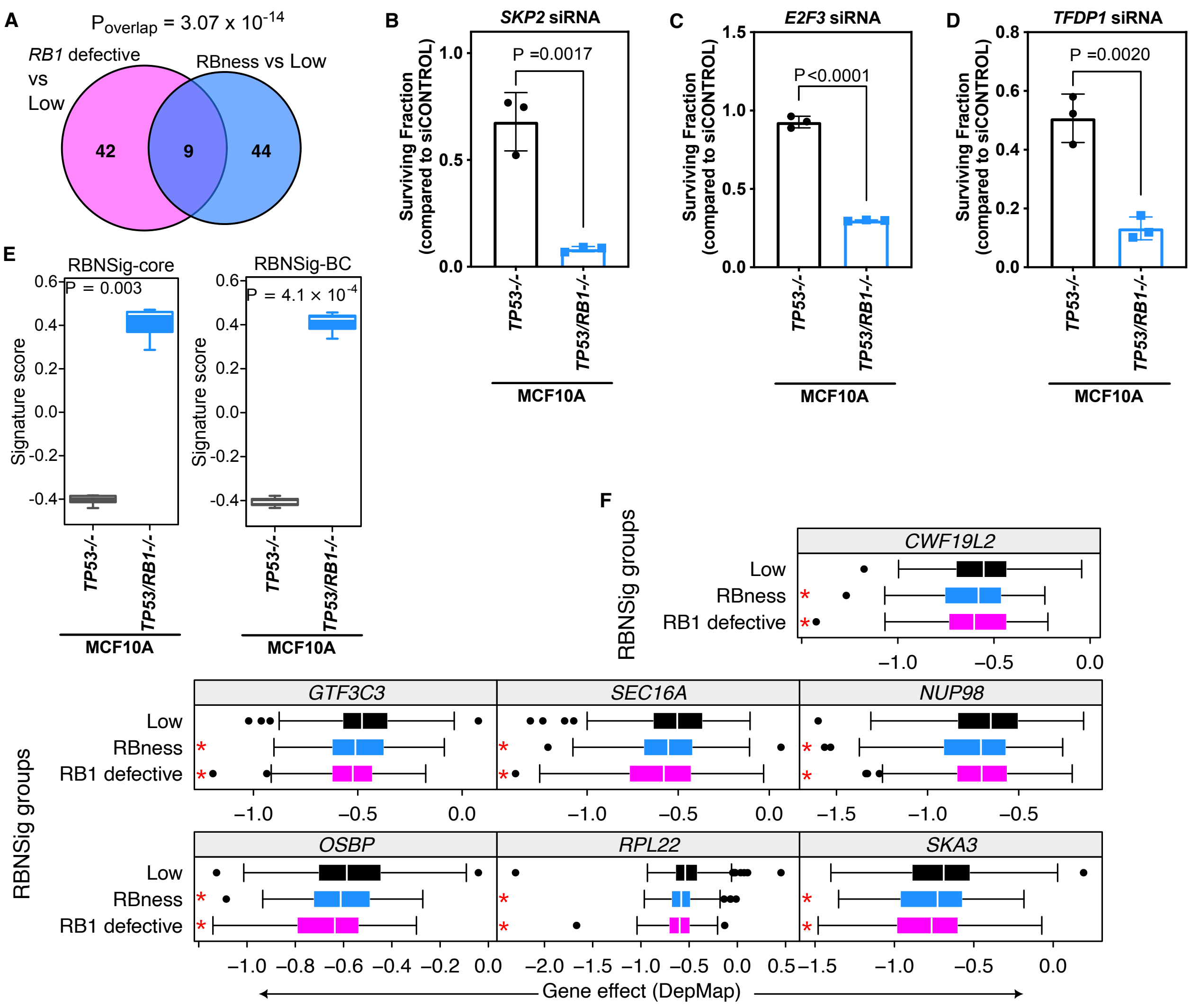

Fig S10. Vulnerabilities and in-vitro validation of RBness and *RB1*-defective cell lines

(A) Venn diagram showing overlap between candidate synthetic lethal genes identified by comparing DepMap's CRISPR-Cas9 gene effect profiles between *RB1*-defective vs RBNSig-low and RBness vs RBNSig-low groups, using generalized linear models adjusted for cell line histology type ($P < 0.05$). Significance of overlap was calculated using the Fisher's exact test. **(B-D)** RNAi knockdown of *SKP2*, *E2F3 and TFDP1* in MCF10A$^{TP53-/-}$ and MCF10A$^{TP53/RB1-/-}$ cells. Viability was assessed 5 days following transfection. Surviving fractions were compared using an unpaired t-test. **(E)** Signature score of RBNSig-core and RBNSig-BC genes using the RNA-Seq data from MCF10A$^{TP53-/-}$ and MCF10A$^{TP53/RB1-/-}$ cells with three replicates each. Signature score was calculated using SVD on TMM normalised followed by $\log_2$(counts per million) transformed data. Unpaired Welch's t-test was used to compare signature scores between the groups. **(F)** DepMap CRISPR-Cas9 gene effect profiles of genes (in addition to the ones shown in Fig. 6D)

that were synthetic lethal with both *RB1* defects and RBness. These were identified in DepMap pan-cancer dataset and validated using the in-house isogenic *RB1* MCF10A $^{TP53/RB1-/-}$ CRISPR-Cas9 screen. Statistically significant genes are shown (‘*’ $P < 0.05$, one-sided Welch’s t-test).

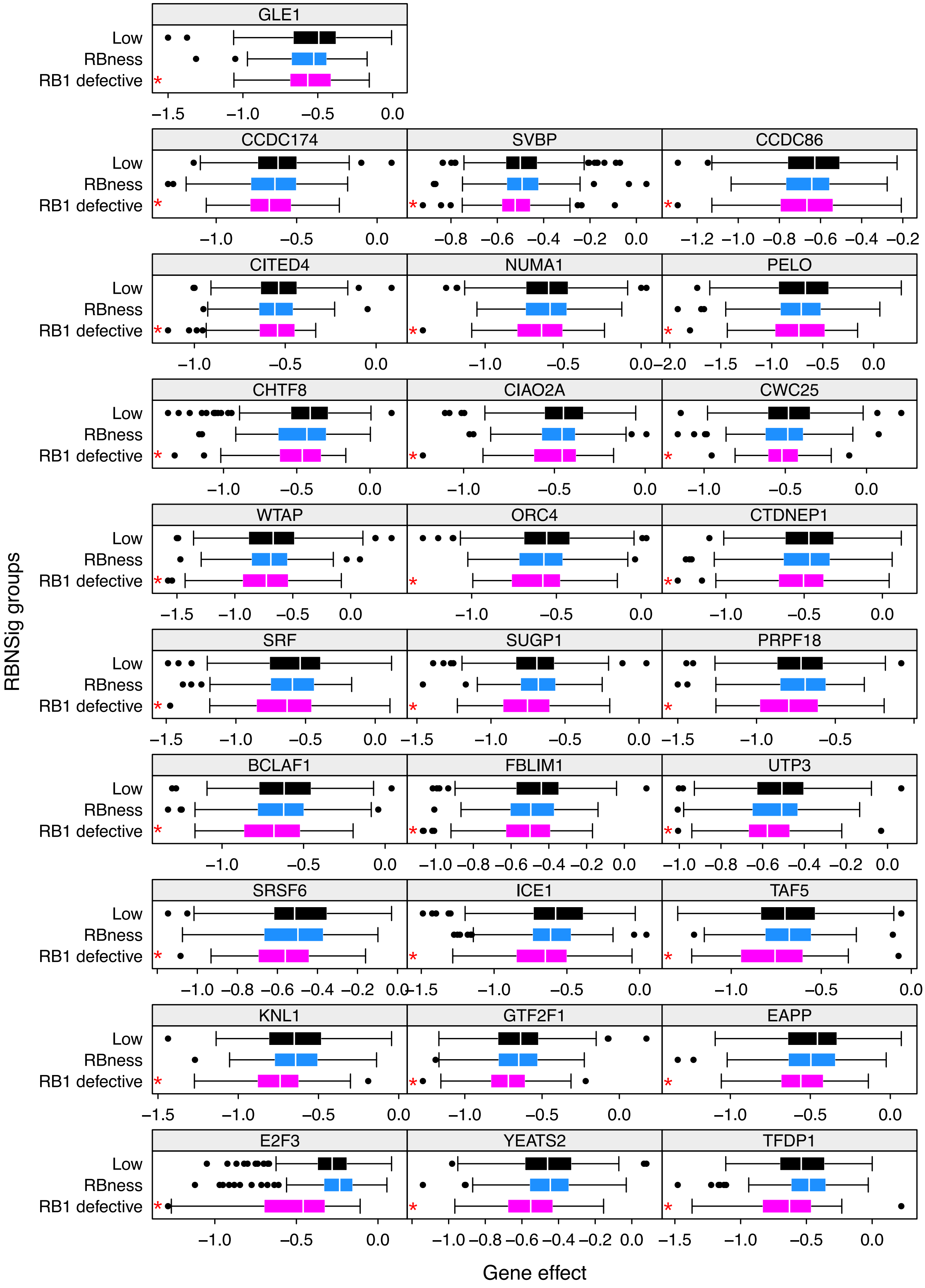
GLE1
CCDC174
SVBP
CCDC86
CITED4
NUMA1
PELO
CHTF8
CIAO2A
CWC25
WTAP
ORC4
CTDNEP1
SRF
SUGP1
PRPF18
BCLAF1
FBLIM1
UTP3
SRSF6
ICE1
TAF5
KNL1
GTF2F1
EAPP
E2F3
YEATS2
TFDP1
Low
RBness
RB1 defective
RBNSig groups
Gene effect

Fig S11. Candidate vulnerabilities of *RB1*-defective cell lines

DepMap CRISPR-Cas9 gene effect profiles of genes that were synthetic lethal with *RB1* defects only. These were identified in DepMap pan-cancer dataset and validated using the in-house isogenic *RB1* MCF10A$^{TP53-/-}$ CRISPR-Cas9 screen. Statistically significant genes are shown ('*' P < 0.05, one-sided Welch's t-test).

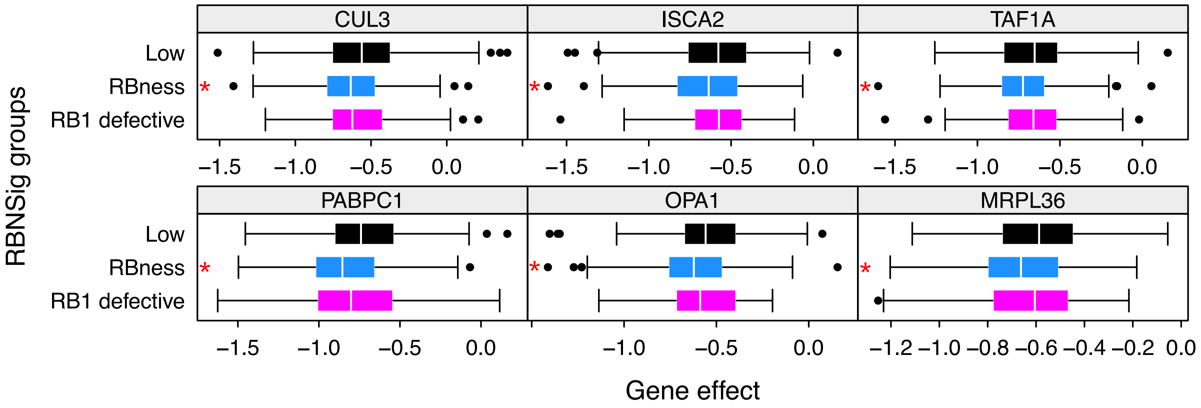

Fig S12. Candidate vulnerabilities of RBness cell lines

DepMap CRISPR-Cas9 gene effect profiles of genes that were synthetic lethal with RBness only. These were identified in DepMap pan-cancer dataset and validated using the in-house isogenic *RB1* MCF10A$^{TP53-/-}$ CRISPR-Cas9 screen. Statistically significant genes are shown ('*' P < 0.05, one-sided Welch's t-test).

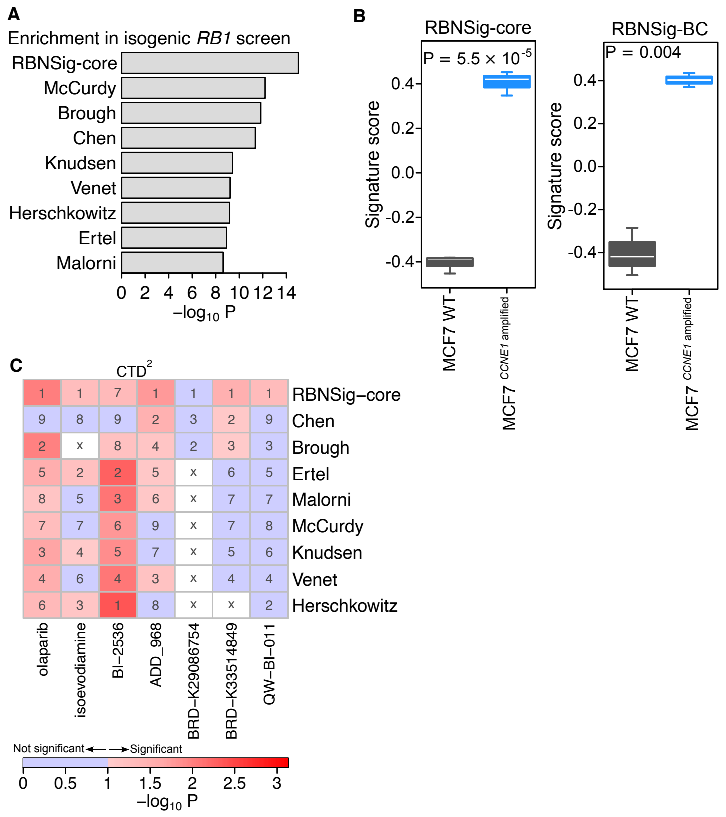

Fig S13. Benchmarking of RBNSig-core in predicting synthetic lethal genes and drug associations, and validation of RBNSig in RBness cell line model

(A) Performance comparison of RBNSig-core against other RB related signatures in detecting synthetic lethal genes. For each signature, synthetic lethal genes were derived by comparing gene effect scores between the signature-high group (cell lines in the top tertile) vs the signature-low group (cell lines in the bottom tertile) using the DepMap dataset. For published signatures, signature score was calculated using SVD. Synthetic lethal genes for each signature were tested for overlap with synthetic lethal genes identified in the isogenic *RB1* MCF10A$^{TP53-/-}$ CRISPR-Cas9 screen using the Fisher's exact test with P values reported in this bar plot. Higher significance indicates greater overlap with the isogenic *RB1* CRISPR screen. **(B)** Signature score of RBNSig-core and RBNSig-BC genes using microarray data (1) from MCF7 and MCF7$^{CCNE1\ amplified}$ cells with three replicates each. Signature score was calculated using SVD on quantile normalised followed by $\log_2$(mRNA abundance) transformed data. Unpaired Welch's t-test was used to compare signature scores between the groups. **(C)** Heatmap showing performance comparison of RBNSig-core and other RB-related signatures in identifying drug associations that were found by RBNSig-core (in Figure 6H). Dose-response AUCs (z-scores) for compounds in CTD^2 that showed

selective sensitivity in RBNSig-core's RBness group (vs low group) were compared to the similar comparison for other signatures' predicted RBness group (vs low group). P values from one-sided Welch's t-test are reported as the background colour and signature's rank (best = 1) is shown as the foreground numbers. 'x' indicates associations that were not assessed due to mean(AUC^{RBness}) > mean(AUC^{Low}).

Table S1

Gene symbols of RBNSig of breast cancer (BC). Column 'low.expressed' contains the genes that are down-regulated (along with *RB1*) in RBNSig-BC-high group and the column 'highly.expressed' contains the genes that are up-regulated in RBNSig-BC-high group.

Table S2

Classification of TCGA BRCA cohort into RBNSig-low (1), no confidence (2) and -high (3) groups using RBNSig-BC.

Table S3

List of STRING network clusters significantly enriched for genes in RBNSig-BC (FDR-adjusted $P < 0.05$).

Table S4

Classification of Metabric cohort into RBNSig-low (1), no confidence (2) and -high (3) groups using RBNSig-BC.

Table S5

Fisher's exact test statistic when RBNSig-BC-high group was stratified into RBness and *RB1*-defective groups and compared for differences in mutation counts, copy-number amplifications and copy-number deletions in MammaSeq^{TM} breast cancer panel (Smith *et al. (35)*). Odd ratio (OR) and P values (P) are reported for mutations (MT), copy-number amplification (AMP) and deletions (DEL) separately. 'NA' represent comparison where >5 patients were not altered and hence deemed unsuitable for statistical comparison.

Table S6

Classification of SCAN-B cohort into RBNSig-low (1), no confidence (2) and -high (3) groups using RBNSig-BC.

Table S7

Predicted alignment scores of RBNSig-BC in BrighTNess cohort.

Table S8

Table of over-representation analysis of RB-related gene signatures in REACTOME pathway database. Over-representation was tested using Fisher's exact test. FDR-adjusted P values reported in this table were adjusted for multiple testing using the Benjamini-Hochberg method.

Table S9

Gene symbols of RBNSig of ovarian cancer (RBNSig-OV). Column 'low.expressed' contains the genes that are down-regulated (along with *RB1*) in RBNSig-OV-high group and the column 'highly.expressed' contains the genes that are up-regulated in RBNSig-OV-high group.

Table S10

Summary of different stages of RBNSig discovery process across 32 cancer types from CPTAC and TCGA datasets. Valid RBNSigs were detected in 14 cancer types (including breast and ovarian cancers).

Table S11

Gene symbols of RBNSigs of 12 cancer types. Column 'low.expressed' contains the genes that are down-regulated (along with *RB1*) in RBNSig-high group and the column 'highly.expressed' contains the genes that are up-regulated in RBNSig-high group. If gene symbol was not available, EntrezID is reported in the format: X<EntrezID>_at

Table S12

log odds (coef) and P value from logistic regression model, adjusted for cancer type. RBNSig-BC-high group was stratified into RBness and *RB1*-defective groups and compared for differences in

mutation counts (MT), copy-number amplifications (AMP) and copy-number deletions (DEL) for each gene in the Cancer Gene Census Tier-1 hallmark drivers. Model was fit to genes where >10 altered samples were available. FDR-adjusted P values (Q) were calculated using the Benjamini-Hochberg method.

Table S13

Gene symbols of RBNSig-core representing recurrently (at least three (20%) cancer types) dysregulated genes with *RB1* across 14 cancer types.

Table S14

List of STRING network clusters significantly enriched for genes in RBNSig-core (FDR-adjusted P < 0.05). Size of the node is proportional to the number of enriched genes, and a link between two clusters exists if there is at least 50% overlap between the sets of enriched genes they included.

Table S15

Classification of DepMap cancer cell lines into RBNSig-low (1), no confidence (2) and -high (3) groups using RBNSig-core. Curated *RB1*-defective cell lines were assigned to '*RB1*-defective' group post-hoc.

Table S16

Statistical comparison of gene effect scores in DepMap aggregated CRISPR-Cas9 loss of function screens, between the *RB1*-defective breast cancer cell lines (g1) and RBNSig-low breast cancer cell lines (g2) using Welch's t-test (one sided, see Methods: CRISPR perturbation screens analysis). FDR-adjusted P values (Q) were calculated using the Benjamini-Hochberg method.

Table S17

Statistical comparison of gene effect scores in DepMap aggregated CRISPR-Cas9 loss of function screens, between the breast cancer cell lines exhibiting RBness (g1, excluding *RB1*-defective cell lines) and RBNSig-low breast cancer cell lines (g2) using Welch's t-test (one sided, see Methods: CRISPR perturbation screens analysis). FDR-adjusted P values (Q) were calculated using the Benjamini-Hochberg method.

Table S18

Statistical comparison of CTD^2 dose response AUC (z-score) of drugs between *RB1*-defective vs RBNSig-low group, and RBness vs RBNSig-low group using Welch's t-test (one sided, see Methods: Association with candidate drugs). P values from generalized linear models (GLMs) adjusted for cell line histology are also reported.
